# The patatin-like lipase family in *Gallus gallus*

**DOI:** 10.1186/1471-2164-9-281

**Published:** 2008-06-12

**Authors:** Jani Saarela, Gerlinde Jung, Marcela Hermann, Johannes Nimpf, Wolfgang J Schneider

**Affiliations:** 1 The Max F. Perutz Laboratories, Department of Medical Biochemistry, Medical University of Vienna, Dr. Bohr Gasse 9/2, A-1030 Vienna, Austria; 2 National Public Health Institute and FIMM, Institute for Molecular Medicine Finland, Haartmaninkatu 8, 00290 Helsinki, Finland

## Abstract

**Background:**

In oviparous species, genes encoding proteins with functions in lipid remodeling, such as specialized lipases, may have evolved to facilitate the assembly and utilization of yolk lipids by the embryo. The mammalian gene family of patatin-like phospholipases (PNPLAs) has received significant attention, but studies in other vertebrates are lacking; thus, we have begun investigations of PNPLA genes in the chicken (*Gallus gallus*).

**Results:**

We scanned the draft chicken genome using human PNPLA sequences, and performed PCR to amplify and sequence orthologous cDNAs. Full-length cDNA sequences of galline *PNPLA2/ATGL, PNPLA4, -7, -8, -9*, and the activator protein *CGI-58*, as well as partial cDNA sequences of avian *PNPLA1, -3*, and *-6 *were obtained. The high degree of sequence identities (~50 to 80%) between the avian and human orthologs suggests conservation of important enzymatic functions. Quantitation by qPCR of the transcript levels of *PNPLA*s and *CGI-58 *in 21 tissues indicates that expression patterns and levels diverge greatly between species. A particularly interesting tissue in which certain PNPLAs may contribute to physiological specialization is the extraembryonic yolk sac.

**Conclusion:**

Knowledge about the exact *in-vivo *functions of PNPLAs in any system is still sparse. Thus, studies about the temporal expression patterns and functions of the enzymes identified here, and of other already known extracellular lipases and co-factors, in the yolk sac and embryonic tissues during embryogenesis are called for. Based on the information obtained, further studies are anticipated to provide important insights of the roles of PNPLAs in the yolk sac and embryo development.

## Background

In egg-laying species, the oocyte's yolk is the major source of nutrients and regulatory factors for the developing embryo. The accumulation of yolk is predominantly the result of massive transport of lipids and proteins from the liver to oocytes situated within ovarian follicles [1]. In mature hens of the domesticated chicken (*Gallus gallus*), fully-grown oocytes contain up to 5 g triacylglycerols (TG) and 240 mg cholesterol, and ovulate every 25 h. In parallel to this oocyte-targeted flow, lipid homeostasis of somatic tissues including the follicular cell layers is maintained. Subsequent to fertilization of the oocyte, normal embryo growth likewise depends on efficient nutrient transfer, i.e., mainly of lipid components, from the yolk into the embryonic circulation. These mobilization processes are maintained through concerted regulation of lipoprotein metabolism involving genes for lipases, lipid transfer proteins, lipoprotein receptors, and others. Thus, the laying hen is a prime model for delineating molecular mechanisms underlying both unidirectional (hepato-oocyte-embryo) mass lipid transfer and fine-tuned regulation of systemic lipid metabolism. Moreover, the known components of this dichotomous regulatory system are highly similar to those in mammals including man, despite the specific tasks they perform [2]. Finally, the laying hen's lipid metabolism is a remarkable paradigm for hormonal control, as estrogen directly regulates not only the hepatic expression of genes for yolk precursor synthesis (e.g., vitellogenin and very low density lipoproteins, VLDL) [3,4], but likely is also involved in the modulation of lipolytic activities and lipid transfer processes important in deposition of yolk into oocytes and subsequent embryonic yolk utilization [5]. Estrogen also induces a unique apolipoprotein residing on VLDL particles, i.e., apoVLDL-II, which inhibits VLDL-lipolysis by lipoprotein lipase (LPL) in the circulation, thereby assuring efficient transport of TG into the oocyte for subsequent use by the growing embryo [6]. Thus, to mediate the mobilization of lipids from yolk, lipolytic enzymes resistant to apoVLDL-II must exist which allow the developing embryo to efficiently access the lipid moiety of yolk lipoproteins.

Yolk mobilization to the embryo proper is mediated by the endodermal endothelial cells of the extraembryonic yolk sac enclosing the yolk during embryogenesis. These cells take up VLDL from the adjacent oocytic yolk via receptor-mediated endocytosis, degrade the particles in lysosomes, and *de novo *synthesize and secrete lipoproteins into the embryonic circulation. The yolk sac-derived VLDL particles subsequently serve as a source of lipid components and of important nutrients in embryo development [7]. Thus, we can define four crucial steps for the ultimate utilization of lipids by the chicken embryo. These steps are, in temporal order: i) massive hormonal induction of yolk precursor synthesis in the maternal liver; ii) oocyte-directed receptor-mediated transport of yolk precursors and their limited proteolysis to form yolk; iii) mobilization of yolk components, mainly lipoproteins, via the yolk sac tissue into the embryonic circulation; and iv) finally, lipid utilization by the tissues of the developing embryo.

The flux of lipid components derived from lipid-carrying macromolecules, from the adult to the yolk, as well as from yolk to the embryo, presumably requires a large number of proteins including a set of lipid hydrolases and regulatory proteins. However, with the exception of the identification of LPL [8,9] and the delineation of circulating apoVLDL-II as inhibitor of LPL [6], to date very little is known about other lipolytic enzymes of the chicken, their genes, expression patterns, modulation and regulation, let alone their functional properties in the above described processes. In man, several patatin-like lipases were identified [10,11], but their physiological roles, with the exception of adiposome TG-lipase (ATGL) [12,13], remain to be elucidated. The patatin-like phospholipases (PNPLAs) contain a patatin domain with lipid acyl hydrolase activity that has been described as nonspecific. Patatin was first identified in potato tubers [14], and subsequently patatin-like phospholipases have been found in pro- and eukaryotes [15]. The patatin domain contains an active site with a serine-aspartate catalytic dyad and an oxyanion hole that stabilizes the enzyme-substrate transition state [16]. The patatin domain shares a conserved core module with mammalian lipases, where the nucleophilic serine is located in a tight turn between a *β*-sheet and an *α*-helix in a well conserved *β*-*β*-*α*-*β *core structure [17] which is also present in, e.g., human cytosolic phospholipase A_2 _[18].

This report provides the first identification, sequence information, and expression profiling of PNPLAs and the associated activator protein (CGI-58) in the chicken. We propose that these newly identified proteins function in processes controlling the distribution and utilization of lipid components within the hepato-oocyte-embryo axis of the chicken.

## Results

### Identification and sequencing of novel chicken genes

To identify novel chicken patatin-like lipases, and the PNPLA2/ATGL-activating protein CGI-58, the draft chicken genomic database was queried using sequences from the human orthologs. The nomenclature of the human PNPLAs as suggested by Wilson *et al*. [19] is adopted throughout this text. PCR was performed to amplify the identified sequences using chicken adipose tissue cDNA as template. The resulting DNA fragments were cloned and sequenced. By this approach we obtained the full-length cDNA clones from the chicken genes corresponding to *PNPLA2/ATGL, PNPLA4, PNPLA8, PNPLA9, PNPLA7*, and *CGI-58*. In addition, we were able to clone partial cDNAs from *PNPLA1, PNPLA3*, and *PNPLA6*. The mRNA sequences of the putative chicken PNPLAs and CGI-58 were deposited in GenBank under accession numbers EU419873 (CGI-58), EU419874 (PNPLA2/ATGL), EU419875 (PNPLA3), EU419876 (PNPLA1), EU419877 (PNPLA4), EU419878 (PNPLA8), EU419879 (PNPLA9), EU419880 (PNPLA6), and EU419881 (PNPLA7).

**Figure 1:**
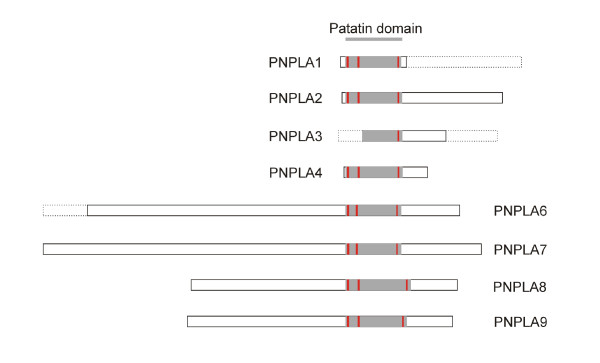
**Schematic representation of the identified chicken patatin-like phospholipases**. The solid lines represent the sequenced patatin-like lipases with N-terminus on the left. The patatin domain is drawn in gray with active site motifs highlighted in red. Thus far non-sequenced parts of the lipases are shown with dashed lines, where the lengths are from the human orthologs. The degrees of identity of the amino acid sequences between the human orthologs and the fully or partially sequenced chicken lipases range from 49% to 83% (for details, see Table 1).

**Figure 2:**
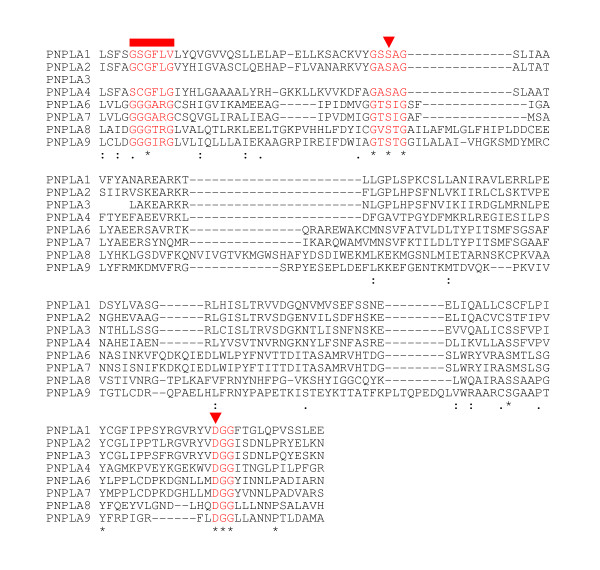
**Patatin domain amino acid sequence alignment of chicken PNPLAs**. Chicken patatin domains were aligned using ClustalW2. Overall, the sequences show little conservation. The active-site motifs, however, are well conserved (shown in red, as in Figure 1). The lipase consensus motif with the catalytic serine, GXSXG, is fully conserved. In addition, the active site aspartate is fully conserved within the consensus DGG-motif. These catalytic dyad residues are indicated by triangles. The valine in the oxyanion hole (the consensus sequence of the motif, (G/A)XGXXG, is indicated by the superscript rectangle) of chicken PNPLA1 corresponds to a serine in the human ortholog. The non-conserved serine (instead of glycine) of the PNPLA4-oxyanion hole is also present in the rat ortholog. The unidentified N-terminal part of PNPLA3 is indicated with blanks. Identical amino acids in all 8 proteins are marked with an asterisk (*), conservative substitutions with a colon (:), and semi-conservative substitutions with a period (.).

The data concerning the newly identified chicken cDNAs and proteins are summarized, together with the available information on human orthologs, in Table 1 (for the sequences, see **Additional file**1). It is apparent that the sizes of the predicted proteins, where full-length clones could be obtained, are very similar to the human proteins, lending support to the assigned designations of the galline lipases. The location of the patatin-like domain within the protein structures predicted from the cloned sequences is shown in Figure 1. In PNPLAs 1–4, the patatin domain is in the N-terminal region, while in PNPLAs 6–9 it is located towards the C-terminus. We were unable to sequence the cDNA encoding the entire patatin domain of *PNPLA3*, but the high degree of similarity of the identified partial cDNA sequence with that of human *PNPLA3*[10] indicates the presence of the avian gene on chicken chromosome 1. The other chicken genes were found on chromosomes 5 (*PNPLA2/ATGL*), 1 (*PNPLA4, -8*, and -*9*), 17 (*PNPLA7*), 26 (*PNPLA1*), and 2 (*CGI-58*). *PNPLA6 *was cloned from an unidentified chromosome. The amino acid alignment of the patatin domains of the eight chicken PNPLAs is shown in Figure 2. The sequence identity among the domains is low, but importantly, the active site motifs are well conserved. On the other hand, the high levels of identity with the human orthologs support the notion that the chicken enzymes identified here have important physiological roles.

**Table 1:** Summary of key features of the patatin-like lipases and CGI-58 from chicken (*Gg*) and man (*Hs*).

**Name(s)**	**Chr. location *Gg*/*Hs***	**Size cDNA (bp) *Gg/Hs***	**Size protein (aa) *Gg/Hs***	**Patatin domain (aa) *Gg/Hs***	**Identity (%) *Gg *vs. *Hs***	**Accession number mRNA *Gg***	**Accession number mRNA *Hs***	**Function(s)**
**PNPLA1**	26/ 6p21	?/ 1602	?/ 533	15–185/ 16–187	~46	EU419876	89886440	?
**PNPLA2** ATGL iPLA2*ζ *desnutrin TTS-2	5/ 11p15.5	1452/ 1515	483/ 504	10–180/ 10–180	67	EU419874	58759050	TG hydrolysis [12], transacylase [10]
**PNPLA3** adiponutrin iPLA2*ε*	1/ 22q13.31	?/ 1446	?/ 481	?/ 10–180	~49	EU419875	17059635	Lipase [11], transacylase [10]
**PNPLA4** iPLA2*η *GS2	1/ Xp22.3	762/ 762	253/ 253	6–177/ 6–177	76	EU419877	458225	Lipase [11], transacylase [10]
**PNPLA5** GS2-like	-/ 22q13	-/ 1290	-/ 429	-/ 12–182	-	-	85397722	Lipase [11]
**PNPLA6** NTE	?/ 19p13.3-13.2	?/ 3984	?/ 1327	?/ 933–1100	~83	EU419880	2982500	Lysophospholipid hydrolysis [37], cell signaling [34]
**PNPLA7** NTE-related esterase NRE	17/ 9q31.3	3975/ 3954	1324/ 1317	936–1103/ 928–1095	73	EU419881	34531027	Esterase [40], Lysophospho-lipase [39]
**PNPLA8** iPLA2*γ*	1/ 7q31	2412/ 2349	803/ 782	465–661/ 445–640	59	EU419878	7670057	Potential lipase [19]
**PNPLA9** PLA2G6 iPLA2*β*	1/ 22q13.1	2391/ 2421	796/ 806	472–657/ 481–666	62	EU419879	3142699	Lipase [29]
**CGI-58** ABHD5	2/ 3p21	1032/ 1050	343/ 349	-	79	EU419873	33469972	Activation of PNPLA2/ATGL [22]

The currently known synonyms for all gene products are listed under Name(s). Chromosomal locations of the genes, size of the cDNAs and proteins (where "?" indicates partial avian sequence), location of the patatin-domain within the proteins, % identities between the chicken and human proteins ("~" is used in cases where partial avian sequence is available), accession numbers of the mRNAs [64], and functions of the proteins where known (functional data from other species are also shown here), are listed with the relevant references. Note that information on the avian PNPLA5 could not be obtained, as described in the text.

PNPLA2/ATGL (also called desnutrin, iPLA2*ζ*, or TTS-2) is the key enzyme for the generation of diglycerides from TG [12,13], and is functionally conserved from yeast to man [20,21]. The enzyme is activated by the protein termed "comparative gene identification 58" (CGI-58, also known as ABHD5) [22]. Deficiency of CGI-58 in man leads to a rare autosomal recessive disease of lipid metabolism, Chanarin-Dorfman syndrome (also called neutral lipid storage disease with ichthyosis; OMIM 275630), characterized by the presence of abnormally large amounts of TG-rich lipid droplets in many tissues [23,24]. Here, we determined that the chicken CGI-58 protein is 79% identical to the human ortholog, the amino acid residues critical for the activation of PNPLA2/ATGL [22], and the putative lipase motif are fully conserved.

PNPLA3 (also known as adiponutrin or iPLA2*ε*) was shown to behave differently from PNPLA2/ATGL with respect to regulation and cellular localization [11,25,26]. PNPLA3 is strongly suppressed upon fasting, but PNPLA2/ATGL expression is increased. Intracellularly, PNPLA3 localizes to membranes, whereas PNPLA2/ATGL is cytosolic.

The human PNPLA family member PNPLA1 has not been fully cloned nor its function studied [11,19]. We were unable to obtain the full-length clone of chicken *PNPLA1 *cDNA, and PCR with several primer pairs was unsuccessful; however, sequence encoding the patatin domain could be obtained. Finally, we could not find any ESTs relevant to *PNPLA1 *in the chicken EST-databases [27,28]. Equally, we could not obtain evidence for the existence of *PNPLA5 *(also termed GS2-like) in the chicken, since the chicken sequence databases available contain no DNA or protein sequence similar to the human or other known orthologs. Immunoprecipitated human PNPLA5 showed lipase activity, and when overexpressed it functioned to decrease intracellular TGs [11].

PNPLA4, also called gene sequence 2 (GS2) or iPLA2*η*, was demonstrated to be expressed in essentially all human tissues and to lipolyze TGs [10]. To date, a murine *PNPLA4 *ortholog has not been reported.

Of the two reportedly unique members of the human patatin-like family [19], PNPLA8 (iPLA2*γ*) is ubiquitously expressed at moderate levels and reported to be transiently upregulated during early differentiation of human adipocytes. The other lipase in this group, PNPLA9, is predominantly expressed in adipose tissue and brain, and is upregulated late during adipocyte differentiation [19]. Endogenous PNPLA9 purified from Chinese hamster ovary cells hydrolyzes dipalmitoyl phosphatidylcholine, and the ankyrin repeats are required for enzyme activity [29].

PNPLA6 (also known as neuropathy target esterase, NTE) is expressed, e.g., in human blood lymphocytes [30] and murine brain [31] and plays a role in membrane lipid homeostasis [32,33]. PNPLA6 was suggested to be involved in a cell-signaling pathway controlling interactions between neurons and glial cells in the developing nervous system [34]. Human [34,35], murine [36,37], and galline [34,38] PNPLA6 were thoroughly studied in relation to their organophosphate-induced inactivation and neuronal degeneration. Finally, rat PNPLA7 (also known as NTE-related esterase or NRE), is highly expressed in prostate, pancreas, and adipose tissue [19], and insulin down-regulates the mouse enzyme, which predominates in testes, skeletal and cardiac muscle, and adipose tissue [39]. The primary sequences of PNPLA6 and -7 have a high degree of identity; their similar features indicate conserved functions. PNPLA7 was suggested to have NTE-related esterase activity [40,41], whereas a more recent study [39] showed that PNPLA7 is a lysophospholipase.

**Figure 3:**
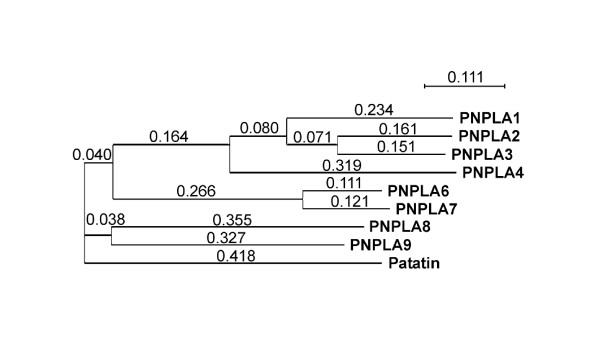
**Phylogram of the chicken PNPLA family**. The phylogram was calculated with ClustalW2 using the chicken patatin domain amino acid sequences. The result shows that PNPLAs 1–4 form a subfamily. PNPLA6 and PNPLA7 also form a subfamily, as do PNPLA8 and PNPLA9. The patatin domains of PNPLA2 and PNPLA3 are 68% identical, of PNPLA1 and PNPLA2 51%, of PNPLA1 and PNPLA3 55%, of PNPLA1 and 4 35%, of PNPLA2 and PNPLA4 37%, of PNPLA3 and PNPLA4 40%, of PNPLA8 and PNPLA9 31%, and of PNPLA6 and PNPLA7 76%. Plant patatin was used as an out-group.

Based on the above results, particularly related to the patatin domains, we analyzed whether the avian homologs also cluster in highly related subgroups, as was reported for the mammalian PNPLAs [19]. The phylogenetic tree shown in Figure 3 indicates that the relationships between the patatin domains of chicken PNPLA2/ATGL, PNPLA1, -3 and -4 are such that they form a distinct subfamily. While in man, PNPLAs 5 and 4 also belong to this family [19], a chicken PNPLA5 homologue could not be identified. A separate subfamily consists of PNPLA6 and -7, and another is made up of PNPLA8 and -9, again in analogy similar to the situation in man [19]. Importantly, the phylogenetic tree generated by analysis of the patatin domain sequences is in agreement with the sizes of the entire proteins and the position of the patatin domains within them (cf. Figures 1 and 3). Furthermore, the residues within the oxyanion hole and the motifs containing the catalytic serine are more highly conserved within the individual (sub)families than between them (Figure 2).

**Figure 4:**
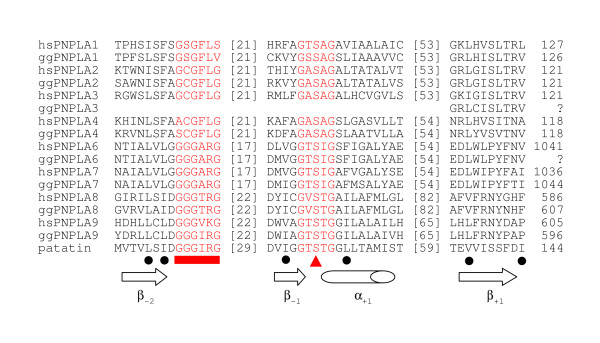
**Comparison of the conserved human and chicken patatin domain core modules**. The GenBank accession numbers for the chicken and human sequences are provided in Table 1. The identities between full-length patatin domains of the human and chicken PNPLA proteins are for PNPLA1, 56%; PNPLA2, 82%; PNPLA4, 74%; PNPLA6, 88%; PNPLA7, 81%; PNPLA8, 84%; PNPLA9, 74%. The partially identified patatin domain of chicken PNPLA3 is 62% identical with the human ortholog. The numbers in brackets between the active site motifs (in red) represent amino acids that are not shown in the alignment. The numbers behind the sequences show the final positions of the displayed sequences with regard to the full-length amino acid sequence. Below the sequences, the triangle indicates the location of the nucleophilic serine, the rectangle indicates the location of the oxyanion hole motif, and dots mark the nonvariant positions of predominantly hydrophobic residues. The 3 open arrows indicate regions folded as *β*-strands, and the cylinder indicates the region folded as *α*-helix in the plant (*Solanum cardiophyllum*) patatin crystal structure (ProteinDataBank entry 1OXW). The positions of these motifs relative to the nucleophilic serine, which is located in a tight turn, are indicated by the subscript numbers.

An alignment of the core modules of galline and human PNPLAs is shown in Figure 4. Within the PNPLA superfamily as now known in these two species (Figure 4), the sequences in the vicinity of the active site motifs are not well conserved, with the exception of the presence of hydrophobic residues in nonvariant positions (indicated in Figure 4). In contrast, the active site motifs are well conserved within subfamilies of the two animal kingdoms. Finally, in all human and galline orthologous PNPLAs, the lengths of the stretches separating the active site motifs are identical.

### Expression profiling

**Figure 5:**
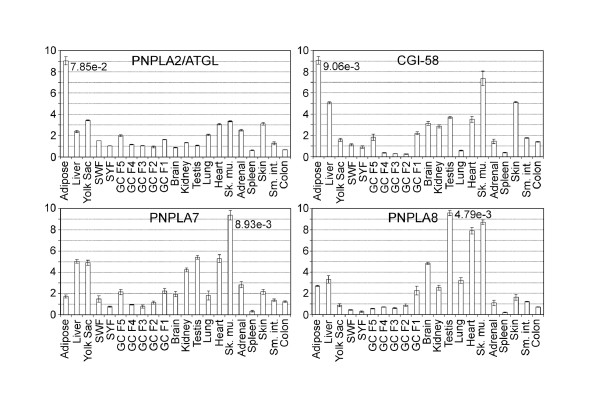
**mRNA expression profiles of chicken PNPLAs and CGI-58**. Gene-specific primers were used to determine the expression profiles of the avian *PNPLAs *and *CGI-58*. Each chicken gene was analyzed twice independently using the LightCycler 480 system. Error bars represent the observed standard deviation. The relative expression values were scaled to a range of 0–10. The values shown were calculated using *β-actin *as the housekeeping gene. For each gene, the highest relative mRNA concentration is shown next to the column representing the corresponding source. Abbreviations: Adip., white adipose tissue; YS, yolk sac from 11 days incubated fertilized egg; SWF, small white follicles; SYF, small yellow follicles; GC 5 – 1, granulosa cells isolated from follicles F5 to F1 (see Methods for details); Sk. mu., skeletal muscle; Adr., adrenal; Sm. int., small intestine.

**Figure 6:**
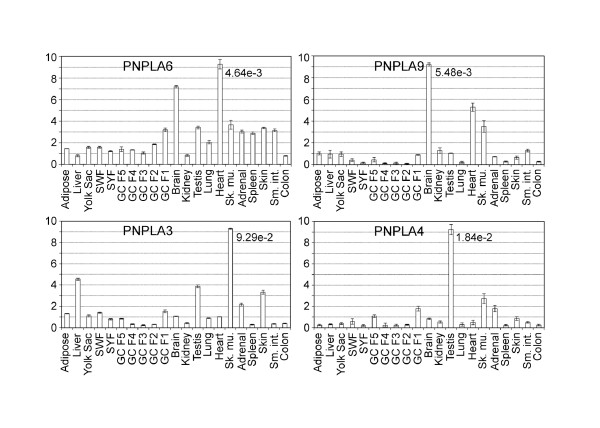
**mRNA expression profiles of chicken PNPLAs**. See Legend for Figure 5 for details.

**Figure 7:**
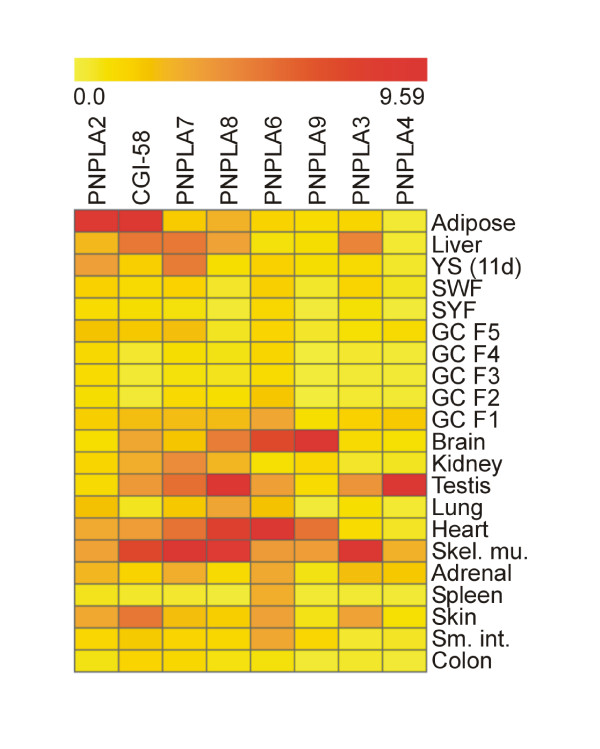
**Heat map showing PNPLA and CGI-58 expression profiles**. The heat map was calculated from the data presented in Figures 5 and 6 using Genesis-software [63]. Red color shows high expression level, while yellow marks low expression level.

To analyze the tissue expression profiles of the above described *PNPLAs*, as well as of *CGI-58*, we measured their mRNA concentrations in 21 chicken tissues or cell types by quantitative real time PCR (qPCR) (Figures 5 and 6). The results show that in tissues highest expression levels of the *PNPLAs *relative to those of *β-actin *span from 4.64e-3 to 9.29e-2. *PNPLA2/ATGL *and its obligatory co-factor, *CGI-58*, show relatively high levels, and are expressed predominantly in adipose tissue. High expression levels of one or both of these genes were also measured in liver, brain, kidney, heart, skeletal muscle, and skin, in addition to yolk sac and testis. The expression patterns of *PNPLA7 *and *PNPLA8 *resemble each other, as do those of *PNPLA6 *and *PNPLA9*. Testis, skeletal muscle, and heart express high levels of *PNPLA7 *and *-8*, whereas *PNPLA6 *and *-9 *transcript levels are high in brain and heart. *PNPLA3 *expression is predominant in skeletal muscle, that of *PNPLA4 *in testis. In the extraembryonic yolk sac, in addition to *PNPLA2/ATGL, PNPLA7 *is expressed at high level. This suggests that at least these two enzymes may perform functions in the yolk sac to mobilize lipid-derived nutrients [7] for utilization by the growing embryo. In Figure 7 the data obtained in the quantitative expression analysis are presented as a heat map to facilitate the evaluation of the relative transcript levels. Generally, in small white and small yellow follicles (containing oocytes which have not yet entered the rapid growth phase; see Methods for details), the expression levels of the genes analyzed are low, and as the oocytes grow, the expression level, particularly of *PNPLA6*, appears to increase in the granulosa cells (Figures 6 and 7; GC F3 to GC F1). In tissues of adult animals, the peak expression levels are seen in white adipose tissue (*PNPLA2/ATGL *and *CGI-58*), brain (*PNPLA9*), testis (*PNPLA8 *and *PNPLA4*), heart (*PNPLA6*), and skeletal muscle (*PNPLA7 *and *PNPLA3*). In addition, high levels compared with most other tissues are present in skeletal muscle (*CGI-58, PNPLA8, -9*, and *-4*), heart (*PNPLA8*, and *-9*), brain (*PNPLA6*), liver (*PNPLA3*), testis (*PNPLA3*), skin (*PNPLA3*), F1 granulosa cells (*PNPLA4*), and adrenal gland (*PNPLA4*).

### *In silico *characterization

The avian proteins (except PNPLA3, due to the incomplete sequence information) were analyzed using *in silico *methods to characterize their properties in more detail. PNPLA2/ATGL contains a predicted nuclear localization signal (VKKAKKKL) at residues 382–389 (the corresponding sequence in human PNPLA2, VMRAKRKL, is not predicted as nuclear localization signal) PNPLA1, -6, -7, -8, and -9 have one or more predicted tyrosine kinase phosphorylation sites. Both PNPLA6 and PNPLA7 contain two putative cyclic nucleotide binding sites indicating that they may be regulated by either cAMP or cGMP. Similar to its human orthologue, chicken PNPLA9 contains several ankyrin repeats, suggesting the potential for protein-protein interactions. The structures of CGI-58 and of all PNPLAs except PNPLA8 are predicted to be globular. Of the fully cloned PNPLAs (PNPLA2/ATGL, PNPLA4, -7, -8, and -9), only PNPLA4 is predicted to contain a signal peptide. PNPLAs 4 and -7 are predicted to contain transmembrane domains at residues 7–26 and 31–53, respectively. Human PNPLA6 was not predicted to contain a membrane spanning domain, but biochemical evidence suggests that its catalytic domain is associated with membranes in neurons [42]. Based on the almost complete identity of the core module sequence to that of human PNPLA6 (Figure 4), this may also be the case for the chicken enzyme. N-glycosylation sites are found in each of the proteins; within both PNPLA2/ATGL and PNPLA4, one N-glycosylation site is located at the N-terminal boundary of the patatin domain, and in PNPLA9 it is close to the C-terminal boundary, respectively.

## Discussion

This is the first study providing insights into the avian patatin domain-containing family of proteins whose mammalian orthologs are known to be lipases that act in several critical physiological processes. The significant degree of sequence conservation for the proposed PNPLAs and CGI-58 proteins in chicken to those of known human proteins lends strong support to the notion that these proteins play critical roles in avian lipid biology. While the catalytic mechanism of most lipases proceeds through the nucleophilic attack of a serine-histidine-aspartate triad [17] and is reminiscent of serine proteases, the patatin family of enzymes operates via a serine-aspartate dyad. The serine of this dyad is located in a so-called nucleophilic elbow, which itself is part of a region suggested by Schneider *et al*. [17] to represent an ancestral sequence module, i.e., one found in both the patatin family (harboring a catalytic dyad) and other lipases, general esterases, and (lyso)phospholipases (operating via a triad). This sequence module, consisting of 50–70 residues forming the *β*_-2 _- *β*_-1 _- *α*_+1 _- *β*_+1 _core (Figure 4), is sometimes interrupted by inserts that may have arisen during evolution [17]. The characteristic nucleophilic elbow of general esterases is located in the loop between the end of the second *β*-strand and the start of the *α*-helix. As shown in Figure 4, the overall structure of the ancestral module clearly is present in the chicken's patatin-like lipases, and the insert lengths are identical between the human and chicken homologue in each of the seven members for which this information could be obtained. Importantly, the active site residues are highly conserved. In addition, in the primary sequence they are flanked by hydrophilic regions.

As revealed by crystal structural analysis, the active site serine-aspartate dyad of the plant patatin [16] and human cytosolic phospholipase A_2 _is located in a deep cleft at the bottom of a funnel [18]. The surface of this funnel is predominantly hydrophobic so as to allow access of fatty acid moieties of substrates. The two glycines in the lipase motif (GXSXG) are structurally essential for the tight turn of the nucleophilic elbow.

As in the murine protein, the protein predicted for the chicken PNPLA2/ATGL contains a potential lipid binding site at residues 311–395 [43]. Importantly, this lipid binding site, unlike other portions of the protein, is as highly conserved as the patatin-like domain. A putative nuclear localization signal (NLS) (predicted at residues 382–389 in the avian sequence) is absent from the human protein, however, and none of the other known PNPLAs are predicted to harbor any NLS; thus, the significance of this observation remains unclear. In man, deficiency of PNPLA2/ATGL causes neutral lipid storage disease with myopathy (OMIM 610717) [44]. Genetic variations within the human *PNPLA2/ATGL *gene influence fasting free fatty acid and glucose levels as well as the risk to develop type 2 diabetes [45]. To date, five mutations in human *PNPLA2/ATGL *have been reported that cause neutral lipid storage disease with myopathy. One is a mutation in which a tetranucleotide duplication leads to a premature truncation of the patatin domain [46]. Fischer *et al*. [44] reported three patients with milder phenotypes, and described four mutant alleles. Three of the mutations are predicted to lead to truncated PNPLA2/ATGL proteins with intact patatin domains, and the fourth leads to a single amino acid substitution (P195L). The affected proline is conserved in human PNPLAs 1–5 and also in chicken PNPLAs 1–4, further indicating an important structural role of the residue.

CGI-58, an activator of PNPLA2/ATGL, belongs to the esterase/thioesterase/lipase subfamily of proteins which are structurally characterized by the presence of *α*/*β*-hydrolase folds. Its interaction with PNPLA2/ATGL is required for TG hydrolysis [22]. An intrinsic lipolytic activity of CGI-58 could not be demonstrated to date [22]; however, CGI-58 may couple lipolytic degradation of cytoplasmic TG to oxidation and packaging into TG-rich lipoproteins for secretion in hepatoma cells [47]. CGI-58 reversibly binds to lipid droplets via perilipin A in the basal state [48,49], whereas PNPLA2/ATGL does not interact with perilipin A [50]. During hormone-stimulated lipolysis, perilipin A phosphorylation by protein kinase A rapidly decreases the interaction between CGI-58 and perilipin A; thus, perilipin A indirectly controls PNPLA2/ATGL activity [50]. The important role of CGI-58 in lipase activation is further underscored by the fact that deficiency of CGI-58 in man [23,24] leads to Chanarin-Dorfman syndrome, characterized by the presence of abnormally large amounts of TG-rich lipid droplets in many tissues.

As in mammals, the predominant expression of chicken PNPLA2/ATGL and CGI-58 in adipose tissue is compatible with the high demand on TG-lipolysis in this tissue. In the chicken, certain tissues such as liver, brain, kidney, heart, skeletal muscle, and skin also show high PNPLA2/ATGL and CGI-58 mRNA abundances, largely in concordance with the sites of TG accumulation in PNPLA2/ATGL deficient patients [44]. In addition, the yolk sac of the chicken embryo shows similarly high mRNA abundances of these genes as well as that of PNPLA7. These observations suggest key roles for the gene products in embryonic yolk mobilization and utilization. PNPLA2/ATGL has been shown to be the main target for activation by CGI-58 [22]. Differences between PNPLA2/ATGL and CGI-58 expression levels may indicate that CGI-58 possibly activates other lipases as well; the mRNA expression level differences might not reflect the protein levels, however, as reported for e.g., subcutaneous and visceral adipose tissue of obese human subjects [51]. In addition, Schweiger *et al*. suggested that mammalian PNPLA2/ATGL likely represents the only lipase activated by CGI-58 [52]. In this context, chicken CGI-58 transcript levels are high in skeletal muscle, where PNPLA3, -7, and -8 also are highly expressed, whereas human PNPLA3 is reported to be expressed exclusively in adipose tissue [44].

The high levels of PNPLA6 and -9 in the brain and of PNPLA4 in testis, respectively, may indicate tissue specific role(s) of these enzymes. In this respect, PNPLA6 is inhibited by organophosphate compounds, leading to delayed neuropathy in man [34] and chicken [31]. Indeed, adult hens are usually used as the animal model for experimental studies of organophosphate-induced delayed neuropathy (OPIDN). The human, murine, and galline PNPLA6s have been reported to hydrolyze a number of lysophospholipids [35-37]. Recently, Chang *et al*. reported partial cloning of the chicken PNPLA6 cDNA [53]. These authors described a novel 3' cDNA sequence to which we add new 5' sequence information and confirm the remaining cDNA sequence reported by them. Human [42] and chicken PNPLA6 do not appear to operate through a conventional catalytic triad, as both proteins contain two aspartate residues, rather than a histidine residue, in addition to the nucleophilic serine. It is interesting to note that while PNPLA6 and -7 are a pair forming one patatin subfamily, and PNPLA8 and -9 form another (cf. Figure 4), expression levels of PNPLA6 and -9 are high in brain, and those of PNPLA7 and -8 in skeletal muscle. This may point to evolution of the two pairs towards performing non-redundant functions in different tissues. In heart, co-expression of PNPLAs 6–9 may indicate the particularly high and complex energy demand of different classes of myocytes.

Currently, we have no evidence that PNPLA5 exists in chicken. We failed to detect any DNA or protein sequence similar to the human or other known orthologs in the chicken sequence databases available. Likewise, we could not find any relevant ESTs in the available chicken EST-databases [27,28]. Lake *et al*. [11] used qPCR to show that PNPLA5 mRNA abundance was greatest in lung, epididymal fat, and brown adipose tissue, but that these expression levels were also ~10000-fold lower than those of PNPLA2/ATGL or PNPLA3 in these same tissues, and undetectable by Northern-blotting [11]. PNPLA5 was induced in the liver of *ob/ob *mice, indicating that the regulation of *PNPLA5 *resembles that of genes involved in hepatic lipogenesis rather than lipolysis [11]. However, it was undetectable in livers of wild-type C57BI/6J mice, and the human ortholog was found almost exclusively in brain [19]. In this context, the human *PNPLA3, PNPLA5 *and *PNPLA9 *loci are syntenic on human chromosome 22q13, and the galline *PNPLA3 *and *PNPLA9 *loci on chromosome 1. Further studies are required to clarify whether galline *PNPLA5 *is syntenic with *PNPLA3 *and *PNPLA9*, but lies in a region with lacking sequence information, or is in fact absent from the entire chicken genome. Finally, our results on galline *PNPLA1 *are in line with the results obtained with *PNPLA1 *from man [22] and mouse [11] in that only partial cDNA sequence could be obtained, and attempts to generate qPCR products, even with several sets of primers, failed. Taken together, the available data suggest that PNPLA1 may not be expressed at detectable levels in any of the three species.

Overall, we note that the expression patterns of several members of the patatin-like lipase family vary significantly in the different species studied to date. For instance, *PNPLA3 *mRNA levels are high in foetal and adult human liver [11], but not in murine liver (Figure 2B in [11]), and is predicted to be high in chicken skeletal muscle (Figure 6), a tissue which in the mouse does not express the enzyme [11]. PNPLA4 shows considerable expression in human heart and skeletal muscle [11], whereas its expression is moderate in chicken heart and skeletal muscle and greatest in the testis (Figure 6). Furthermore, PNPLA5 expression is high in human brain [11], but not in murine brain [11], whereas expression is high in the lung of mice [11], but not of man [11]. On the other hand, a notable exception to these examples is PNPLA2/ATGL, whose expression in adipose tissue consistently prevails over that in other tissues in all species. Since preliminary results (J. Schatte and A. Hermetter, personal communication) indicate that chicken PNPLA2/ATGL indeed possesses TG-lipase activity that is enhanced by CGI-58, it is likely that also the other PNPLA genes have important *in-vivo *roles in the chicken. Another tissue with significant expression of *PNPLA2/ATGL *(and of *PNPLA7*) is the yolk sac of the avian embryo, where lipases likely have significant functions in the mobilization of lipid components from yolk for utilization by the embryo. In this context, Kienesberger *et al*. [39] have provided strong evidence that murine PNPLA7 is a lysophospholipase that is regulated by nutritional status and insulin.

Major transformations in yolk lipid composition occur during the transport across the yolk sac, an extraembryonic structure consisting of an outer mesoderm of flattened support cells and an inner layer of endodermal endothelial cells (EECs). The EECs are responsible for yolk absorption and secretion of *de novo *synthesized lipids and lipoproteins into capillaries emanating from villi on the basal surface of the EECs [5]. Thus, the chick embryo's yolk sac functions in lipid transport highly analogous to the mammalian placenta in terms of nutrient provision to the embryo, and/or to the processes of intestinal lipid absorption and secretion. Following uptake of lipids into the yolk sac, almost all unesterified yolk cholesterol (80% of total cholesterol) is esterified, which then amounts to 81% of the total cholesterol in the yolk sac. One striking observation associated with lipid metabolism in the avian embryo is that the proportions of 20:4n-6 and 22:6n-3 fatty acids progressively increase during transit from yolk to the embryonic plasma. Lipolytic and re-esterification processes involving lipases lead to the almost exclusive presence of 22:6n-3 fatty acid in the phospholipid fraction of yolk, but interestingly, 22:6n-3 is subsequently present in the embryonic plasma's TG-rich lipoproteins, which originate in the EECs. There is an almost 60-fold enrichment of 22:6n-3 from yolk TG to plasma TG [54]; 20:4n-6 preferentially distributes to phospholipids in yolk and embryonic plasma, with much smaller changes in concentrations between the fractions than observed for 22:6n-3. These and other observations [5] are compatible with the involvement of multiple hydrolytic activities in cholesterol esterification, desaturation/elongation reactions, translocation of 22:6n-3 from phospholipids to TG, and in the overall conversion of yolk lipid components to plasma lipoproteins that supply the developing embryonic organs via specific uptake mechanisms.

Future studies will address the detailed temporal expression patterns of the patatin-like putative lipases identified, and of other already known extracellular lipases and co-factors, in the yolk sac and the embryonic tissues during embryonic development. Knowledge about the exact *in-vivo *functions of these enzymes in any system is still sparse. Based on the information gained here, it is reasonable to anticipate important insights of general relevance through exploitation of the advantages of the chick system [55,56].

## Conclusion

Data mining of the draft genome of *Gallus gallus *combined with cDNA cloning and tissue expression analysis by quantitative PCR have resulted in the first identification of hitherto unknown putative lipolytic enzymes in this powerful experimental system. Comparison of findings in birds, which split from the mammalian lineage about 310 million years ago, with those in mammals provides additional analytical power. This fact is demonstrated here for the gene family encoding patatin domain-containing proteins which putatively function as lipases. Future studies in the chicken may reveal important features shared by this gene family and their products in different animal phyla.

## Methods

### Animals and tissue samples

Derco white laying hens (30–40 weeks old) and roosters were purchased from Heindl Co. (Vienna, Austria) and maintained on layer's mash with free access to water and feed under a daily light period of 14 h. Fertilized chicken eggs were incubated under standard conditions for temperature (38°C) and humidity (70%). The eggs were removed from the incubator after 11 days of incubation, and the shell was cut to expose the yolk sac membrane. The yolk sac membranes were excised and washed with ice-cold phosphate-buffered saline (PBS) and subsequently used for RNA extraction as described below. The preovulatory small white (less than 5-mm diameter, without yolk) and small yellow follicles (5–12-mm diameter, with yolk) form a pool from which the hierarchical follicles (F1-F7) develop. The F1 follicle is the largest and most mature follicle (to be ovulated on collection day), while higher numbered follicles are progressively smaller and less mature. Granulosa cells were isolated from F5 – F1 follicles and washed with PBS [57]. Other chicken tissue samples were immediately frozen in liquid nitrogen after collection of samples or used for RNA extraction. White adipose tissue was obtained from the abdominal cavity.

### Total RNA extraction, cDNA synthesis, and sequencing

Chicken total RNA was extracted from tissue using Nucleospin RNA II -kit (Macherey-Nagel) according to instructions from manufacturer. When tissue samples were not immediately used for RNA extraction, lysis buffer with chaotropic ions was added prior to freezing to stabilize RNA. All tissues used were collected from mature hens except testis, which was from mature roosters. Yolk sac and granulosa cells were prepared as described above. Single-stranded cDNA was synthesized using SuperScript II reverse transcriptase (Invitrogen) and Oligo(dT)_10 _-primer. Patatin-like lipase genes were searched for in the draft chicken genomic sequence database [58]. Several primer pairs were designed to produce overlapping fragments, and finally the pairs shown in Table 2 were used to clone the indicated cDNAs. PCR amplification was performed with a T3000 Thermocycler (Biometra) with touch-down program using Phusion Hot Start- (Finnzymes), DyNAzyme EXT- (Finnzymes), or PfuTurbo C_x _Hotstart -(Stratagene) DNA polymerase with chicken adipose tissue cDNA as template. In touch-down PCR, the following conditions were used during the amplification cycles: 3 cycles at annealing temperature 3°C above the melting temperature of the primer with lower value; 3 cycles at the melting temperature; 34 cycles at 3°C below the melting temperature. Other steps of the PCR were as follows: +95°C for 2 min; cycles: +95°C for 30 s, annealing for 30 s, +72°C for 1 min/kb; +72°C for 10 min. The amplified products were subjected to agarose gel electrophoresis (1%) and stained with ethidium bromide. Subsequently, the PCR product was excised from the gel and DNA was purified with QIAquick Gel Extraction Kit (Qiagen). If Phusion or PfuTurbo polymerase was used in the PCR, a tailing reaction was performed. 7 *μ*l purified PCR product was incubated for 30 min at +70°C with 1 *μ*l2 mM dATP, 1 *μ*l 10× PCR buffer, and 1 *μ*l (1 U/ml) Taq DNA polymerase (Roche). Next, the DNA was cloned into the pCR2.1 TOPO -vector with the TOPO TA Cloning -kit (Invitrogen), transformed into the chemically competent TOP10F *E. coli *bacterial strain (Invitrogen), and plated on Luria-Bertani plates containing 50 *μ*g/ml kanamycin and 40 *μ*g/ml X-gal (5-bromo-4-chloro-3-indolyl-b-D-galactoside). Insert-containing bacterial clones were screened with colony PCR using the above described PCR conditions and primers. Positive clones were incubated overnight +37°C in Luria-Bertani medium containing 50 *μ*g/ml ampicillin, and plasmid DNA was purified with FastPlasmid Mini -kit (Eppendorf). DNA sequencing was performed by VBC-Biotech Service GmbH (Vienna, Austria).

**Table 2:** Primers used for the cloning of chicken cDNAs.

**Gene**	**Forward/Reverse**	**Sequence (5'-3')**	**Melting temperature (°C)**
*PNPLA1*	Forward	ATGGCTGCAGAGGAGCTGCG	64
*PNPLA1*	Reverse	TTCCTCCAGGCTGGAGACGG	64
*PNPLA2*	Forward	ATGTTCCCTTTGGACTCCGC	59
*PNPLA2*	Reverse	TCAGAAGAGTGGCAGGCACTC	62
*PNPLA3*	Forward	TGGCCAAAGAAGCTAGAAAGCG	60
*PNPLA3*	Reverse	AAGCCTAGGAGGTAGACTCTC	60
*PNPLA4*	Forward	ATGAAACGTGTCAATCTATCATTTG	56
*PNPLA4*	Reverse	CACAGCTATAGGCATTCTGCC	60
*PNPLA6*	Forward	GTGTTGGGACACTTTGAGAAGC	60
*PNPLA6*	Reverse	ATAGATCTCATCGAACTTGC	53
*PNPLA7*	Forward	ATGGAAGAAGAAGGCAACGGCACC	64
*PNPLA7*	Reverse	CTAGGCTGGCTCTCCTGTGCTGC	68
*PNPLA8*	Forward	ATGACAGTTCATTTGTCTCTAG	55
*PNPLA8*	Reverse	GCGTTACGTATTCACAATTTGG	57
*PNPLA9*	Forward	ATGCAGTTCCTCGGGCGGCTTTTG	66
*PNPLA9*	Reverse	TCATCGGCAGAGATACTGCACCAG	64
*CGI-58*	Forward	ATGGCCGAGGAGGAGGCCTCCAG	70
*CGI-58*	Reverse	TCAGTCCACAGAATCACAGATG	58

### Nucleotide sequence accession numbers

The mRNA sequences of the chicken PNPLAs and CGI-58 were deposited in GenBank under accession numbers: EU419873 for CGI-58, EU419874 for PNPLA2/ATGL, EU419875 for PNPLA3, EU419876 for PNPLA1, EU419877 for PNPLA4, EU419878 for PNPLA8, EU419879 for PNPLA9, EU419880 for PNPLA6, and EU419881 for PNPLA7.

### Quantitative real time PCR

Quantitative real time PCR (qPCR) was performed with the LightCycler 480 system (Roche) using LightCycler FastStart DNA Master SYBR Green I -kit (Roche) according to the manufacturer's instructions. The qPCR conditions were as follows: +98°C for 10 min; 45 amplification cycles at +98°C for 10 s, annealing for 10 s (5°C above melting temperature of the primer with lower value), elongation at +72°C for 14 to 17 s (25 bp/s + 2 s), measurement of fluorescence at +79–91°C for 1 s (see Table 3). After amplification, melting curve analysis was performed as follows: +98°C for 10 s; annealing temperature for 20 s; continuous temperature gradient to +98°C with 5 acquisitions/s. The tissue cDNA samples (from adult animals and the indicated tissues and cells) were diluted 1/20 with water, and the same cDNA dilution was used for all qPCR reactions. A fresh 10^-1 ^to 10^-9 ^serial dilution was prepared from each target gene to be analyzed starting from the 1/20 dilution of a purified PCR product, and every gene was measured twice independently with duplicate samples. From the serial dilution, 10^-4 ^to 10^-8 ^dilutions were used as an internal standard. As housekeeping genes, we measured the mRNA expression levels of chicken *β-actin*, *5-aminolevuline synthase (ALAS*), and *porphobilinogen deaminase (PBGD*). The qPCR primers used are shown in Table 3. In qPCR, every gene had PCR efficiencies ranging from 1.926 to 1.972, as calculated from the internal standard; thus, no efficiency correlation was performed. The LightCycler 480 Software (release 1.1.0.0520) with absolute quantification was used to calculate the concentrations and standard deviations of the samples. The concentration of the target gene was divided by the concentration of the housekeeping gene thus showing the relative expression of the mRNA in the tissue. This calculation was performed for all tissues analyzed. Standard deviations were calculated by multiplying the obtained relative value with the standard deviation of the target. The resulting relative and standard deviation values were scaled to a range of 0–10 including the standard deviations. Quantitative PCR was not successful with *PNPLA1*. Specificity of the amplification reactions was confirmed by sequencing of the qPCR products.

**Table 3:** Primers used for the qPCR analyses.

**Gene**	**Forward/Reverse**	**Sequence (5'-3')**	**Melting temperature (°C); Temperature of fluorescence measurement (°C)**
*PNPLA2*	Forward	CAGCACCTTTATCCCTGTCTAC	60; 83
*PNPLA2*	Reverse	AATTGGATGCTTGTATTGGTG	54
*PNPLA3*	Forward	GTTGTCCAGGCATTGATCTG	57; 82
*PNPLA3*	Reverse	ATCACACTCCCCAGCAAAAG	57
*PNPLA4*	Forward	ATGCTGGAATGAAGCCAGTC	57; 79
*PNPLA4*	Reverse	CTGGTTTGGTGGAAACATAGC	58
*PNPLA6*	Forward	CACCGTTGAGTGGCTCAACATG	62; 91
*PNPLA6*	Reverse	CTCCTCCATCGCCTTGATCAC	62
*PNPLA7*	Forward	TGTTCCAGAAGCCACCAGAC	59; 86
*PNPLA7*	Reverse	CTGCGTTCTTCTGCGTACAG	59
*PNPLA8*	Forward	ACTGTGGCAGGCCATTAGAG	59; 84
*PNPLA8*	Reverse	TTTCATATCGGCCAGTACCC	57
*PNPLA9*	Forward	AGGACATGGTTCGGTCTCTG	59; 88
*PNPLA9*	Reverse	GCGTCATCTCTGCCTTCTTC	59
*CGI-58*	Forward	GATTCCTGGCTGCTGCTTAC	59; 82
*CGI-58*	Reverse	CGCTGAACAAGGCTTAGACC	59
*β-actin*	Forward	AGCTATGAACTCCCTGATGG	57; 84
*β-actin*	Reverse	ATCTCCTTCTGCATCCTGTC	57
*ALAS*	Forward	GGCAGCTCAGATGAACCACAAG	62; 82
*ALAS*	Reverse	CGGGTCTTTGCTTCAGCATAATATC	61
*PBGD*	Forward	AACTGTGGGAAAACGCTCAG	57; 82
PBGD	Reverse	GGCTCAGGAGCTGACCTATG	61

### *In silico* characterization

The obtained sequences were analyzed using the PredictProtein [59], and SignalP [60]. Multiple sequence alignment and phylogenetic tree calculation were performed using ClustalW2 [61]. Transmembrane helices were predicted using TMHMM 2.0 [62].

## Abbreviations

TG: Triacylglycerol; PNPLA: Patatin-like phospholipase; iPLA2: Calcium-independent phospholipase A2; qPCR: Quantitative real time PCR; CGI-58: Comparative gene identification 58; VLDL: very low density lipoprotein; LPL: lipoprotein lipase; ATGL: adiposome triglyceride lipase; GC: granulosa cell; EEC: endodermal endothelial cell.

## Authors' contributions

WJS designed the study, participated in the interpretation of the data, advised in the design of data presentation, and contributed to the final version of the manuscript. JS participated in the study design, performed the experiments with the assistance of GJ, contributed to the interpretation of the data, and wrote the manuscript draft. MH provided the preparations of tissues and cells used in the study. JN contributed to the design and interpretation of the data and items to be included in the Discussion. All authors made suggestions to drafts, and read and approved the final manuscript.

## Supplementary Material

Additional file 1 Click here for file
