# Supplementary material for: The patatin-like lipase family in Gallus gallus
Source: BMC Genomics. 2008 Jun 12;9:281. doi: 10.1186/1471-2164-9-281 (PMC2435558; doi:10.1186/1471-2164-9-281)
Supplement: Additional file 1 [file 1471-2164-9-281-S1.pdf]

Additional data file 1 - Chicken PNPLA and CGI-58 sequences.

cDNA and amino acid sequences of the identified chicken genes and their GenBank accession numbers. Start and stop codons are underlined.

PNPLA1 (partial), cDNA: EU419876, protein: ACC91736

```
  1   M  A  A  E  E  L  R  A  P  G  T  P  F  S  L  S  F  S  G  S
  1  ATGGCTGCAGAGGAGCTGCGTGCTCCGGGCACCCCTTTCTCGCTCTCCTTTTCGGGCAGT

 21   G  F  L  V  L  Y  Q  V  G  V  V  Q  S  L  L  E  L  A  P  E
 61  GGGTTCCTCGTGCTGTATCAGGTTGGGGTGGTGCAGTCCCTCCTGGAGTTGGCCCCCGAG

 41   L  L  K  S  A  C  K  V  Y  G  S  S  A  G  S  L  I  A  A  A
121  CTGCTCAAATCTGCCTGCAAGGTGTACGGATCGTCTGCCGGATCACTCATCGCCGCCGCC

 61   V  V  C  G  I  S  L  D  D  L  K  E  V  F  Y  A  N  A  R  E
181  GTGGTGTGCGGCATCAGCCTGGATGACCTGAAAGAAGTGTTCTATGCAAACGCAAGGGAA

 81   A  R  K  T  L  L  G  P  L  S  P  K  C  S  L  L  A  N  I  R
241  GCCCGGAAAACCCTGCTGGGCCCCCTGTCCCCCAAGTGCAGCCTGCTGGCCAACATCAGG

101   A  V  L  E  R  R  L  P  E  D  S  Y  L  V  A  S  G  R  L  H
301  GCCGTGCTGGAGCGCAGGCTCCCGGAGGACTCCTACCTGGTGGCATCGGGGCGGCTGCAC

121   I  S  L  T  R  V  V  D  G  Q  N  V  M  V  S  E  F  S  S  N
361  ATCTCTCTGACGCGGGTGGTGGACGGCCAGAACGTCATGGTGTCCGAGTTCAGCTCGAAT

141   E  E  L  I  Q  A  L  L  C  S  C  F  L  P  I  Y  C  G  F  I
421  GAGGAGCTCATTCAGGCTCTCCTCTGTAGCTGCTTCCTTCCTATCTACTGCGGGTTCATC

161   P  P  S  Y  R  G  V  R  Y  V  D  G  G  F  T  G  L  Q  P  V
481  CCTCCGTCCTACCGAGGTGTGCGTTACGTTGATGGGGGATTCACGGGCCTGCAGCCCGTC

181   S  S  L  E  E
541  TCCAGCCTGGAGGAA
```

PNPLA2/ATGL, cDNA: EU419874, protein: ACC91734

```
  1   M  F  P  L  D  S  A  W  N  I  S  F  A  G  C  G  F  L  G  V
  1  ATGTTCCCTTTGGACTCCGCTTGGAACATCTCCTTCGCCGGCTGCGGCTTCCTAGGGGTC

 21   Y  H  I  G  V  A  S  C  L  Q  E  H  A  P  F  L  V  A  N  A
 61  TACCACATCGGCGTGGCCAGCTGTCTCCAGGAGCATGCTCCTTTCCTCGTCGCCAACGCC

 41   R  K  V  Y  G  A  S  A  G  A  L  T  A  T  A  L  V  S  G  A
121  CGGAAGGTGTACGGGGCCTCGGCCGGCGCGCTCACCGCCACCGCCCTCGTCAGCGGCGCC

 61   C  L  G  E  A  G  A  S  I  I  R  V  S  K  E  A  R  K  R  F
181  TGCCTCGGTGAGGCTGGTGCCAGCATTATTCGAGTGTCCAAAGAAGCACGAAAGAGGTTC

 81   L  G  P  L  H  P  S  F  N  L  V  K  I  I  R  L  C  L  S  K
241  CTGGGTCCGCTTCACCCCTCTTTCAACTTGGTGAAGATCATTCGGCTCTGCCTGTCCAAG

101   T  V  P  E  N  G  H  E  V  A  A  G  R  L  G  I  S  L  T  R
301  ACTGTGCCAGAGAATGGGCATGAGGTGGCAGCAGGACGTTTGGGTATTTCCCTCACACGA

121   V  S  D  G  E  N  V  I  L  S  D  F  H  S  K  E  E  L  I  Q
361  GTGTCTGATGGAGAAAACGTCATACTATCAGACTTCCATTCCAAGGAGGAGCTGATCCAG
```

```
 141   A  C  V  C  S  T  F  I  P  V  Y  C  G  L  I  P  P  T  L  R
 421  GCCTGTGTCTGCAGCACCTTTATCCCTGTCTACTGTGGGCTGATACCTCCAACTTTGCGT

 161   G  V  R  Y  V  D  G  G  I  S  D  N  L  P  R  Y  E  L  K  N
 481  GGAGTGAGATATGTTGATGGAGGAATTTCTGACAACTTGCCACGATATGAGCTGAAGAAC

 181   T  I  T  V  S  P  F  S  G  E  S  D  I  C  P  R  D  S  S  T
 541  ACAATCACGGTGTCTCCGTTCTCAGGAGAGAGTGATATCTGCCCACGAGACAGTTCCACA

 201   N  M  H  E  L  R  V  T  N  T  S  I  Q  F  N  L  R  N  L  Y
 601  AACATGCATGAGCTGAGAGTCACCAATACAAGCATCCAATTCAACCTTCGCAACCTCTAC

 221   R  L  S  K  A  L  F  P  P  E  P  Q  V  L  R  D  M  C  K  Q
 661  CGCCTCTCAAAGGCCCTCTTTCCTCCAGAGCCACAGGTGCTGCGGGATATGTGCAAGCAG

 241   G  Y  R  D  A  L  H  F  L  K  K  N  G  L  L  H  L  Q  R  P
 721  GGCTATCGGGATGCGCTGCACTTCCTGAAGAAGAATGGTCTCCTTCACCTTCAGCGTCCA

 261   N  P  A  G  P  L  L  A  I  E  A  P  P  G  E  K  K  E  E  E
 781  AATCCTGCTGGTCCTCTCCTTGCAATAGAAGCCCCTCCAGGAGAGAAGAAAGAGGAAGAA

 281   K  E  A  E  D  Q  M  E  D  N  T  A  L  A  V  V  E  D  H  I
 841  AAAGAAGCTGAGGACCAGATGGAGGACAACACTGCCCTTGCAGTTGTGGAAGACCACATC

 301   F  E  H  L  P  P  K  L  N  Q  A  L  L  E  A  C  A  E  R  R
 901  TTCGAACACTTGCCTCCCAAACTGAACCAAGCTCTTTTGGAGGCATGTGCTGAAAGAAGA

 321   G  L  L  S  G  I  S  N  T  L  P  I  R  V  A  T  A  M  M  V
 961  GGTCTCTTGAGTGGTATTAGCAACACGCTGCCTATACGTGTGGCCACTGCCATGATGGTC

 341   P  Y  L  L  P  L  E  S  A  V  S  F  T  V  R  L  L  E  W  L
1021  CCCTACCTGCTGCCTCTGGAGTCTGCTGTTTCCTTCACTGTCAGATTGCTGGAATGGCTT

 361   P  D  I  P  E  D  I  R  W  M  R  E  Q  I  I  E  I  C  N  Y
1081  CCAGATATCCCTGAGGATATTAGATGGATGAGGGAGCAGATAATTGAAATCTGCAACTAT

 381   L  V  K  K  A  K  K  K  L  G  S  H  L  S  A  R  L  Y  Y  H
1141  CTTGTGAAGAAAGCCAAGAAGAAACTGGGCAGTCATCTTTCAGCCAGGCTTTACTATCAC

 401   L  E  L  G  G  P  Q  S  L  P  I  S  S  A  S  P  C  G  E  A
1201  CTCGAGCTTGGAGGGCCCCAGAGCCTGCCAATTTCTTCAGCATCTCCTTGTGGCGAGGCA

 421   M  P  M  W  M  R  S  N  R  S  L  S  D  V  M  L  K  W  E  E
1261  ATGCCTATGTGGATGAGAAGCAACCGTTCCCTGTCTGATGTCATGCTGAAGTGGGAGGAG

 441   Y  Q  R  Q  L  V  M  G  L  L  C  I  N  V  D  M  Q  A  S  L
1321  TACCAGCGTCAGCTTGTGATGGGCTTGCTTTGCATCAACGTGGACATGCAGGCATCCCTC

 461   F  P  W  E  G  F  Q  M  K  L  P  P  L  D  C  A  K  E  C  L
1381  TTCCCTTGGGAAGGATTTCAGATGAAACTTCCACCTCTGGACTGTGCAAAAGAGTGCCTG

 481   P  L  F  -
1441  CCACTCTTCTGA
```

PNPLA3 (partial), cDNA: EU419875, protein: ACC91735

```
   1   E  A  R  K  R  N  L  G  P  L  H  P  S  F  N  V  I  K  I  I
   1  GAAGCTAGAAAGCGGAATCTTGGTCCTCTTCATCCTTCATTTAATGTGATAAAGATAATA

  21   R  D  G  L  M  R  N  L  P  E  N  T  H  L  L  S  S  G  R  L
  61  AGAGATGGATTGATGAGAAATCTGCCAGAAAACACTCACCTGTTGTCATCGGGCAGGCTG

  41   C  I  S  L  T  R  V  S  D  G  K  N  T  L  I  S  N  F  N  S
```

121 TGCATTTCACTGACTAGAGTCTCAGATGGTAAAAACACACTGATATCTAATTTTAACTCT

61 K E E V V Q A L I C S S F V P I Y C G L
181 AAAGAAGAAGTTGTCCAGGCATTGATCTGTAGCTCATTTGTCCCAATTTATTGTGGCCTC

81 I P P S F R G V R Y V D G G I S D N L P
241 ATTCCGCCATCATTTAGAGGTGTGCGCTACGTGGATGGAGGAATCAGTGACAACTTGCCT

101 Q Y E S K N T I T V S P F A G E C D I C
301 CAGTATGAATCTAAGAATACCATCACCGTTTCACCTTTTGCTGGGGAGTGTGATATCTGT

121 P K G N S A N F H E M N V T N T S I Q L
361 CCAAAGGGGAATTCTGCCAACTTTCATGAAATGAATGTGACCAACACCAGCATTCAGCTC

141 S L G N L Y R L T Q A L F P P E P K V L
421 AGTTTGGGGAACCTTTATCGTTTAACACAAGCACTCTTTCCTCCAGAACCTAAGGTACTA

161 G E I C E Q G Y S D A F K F L K E N G I
481 GGTGAGATCTGTGAGCAAGGATATTCAGATGCTTTTAAATTCCTGAAAGAGAATGGTATT

181 L N D S I Y V S L S F T K T N P H E A A
541 CTGAATGACTCAATTTATGTCAGCTTGTCCTTCACCAAAACAAATCCTCATGAAGCTGCA

201 Q H I D Y M K K R N K S E N S R M E T S
601 CAACACATTGACTATATGAAGAAAAGAAATAAGTCTGAAAACAGTAGAATGGAAACTTCA

221 Q V E V L G E Q M K Q N P W P L E K S I
661 CAAGTGGAAGTACTCGGTGAGCAGATGAAGCAAAATCCGTGGCCTCTGGAGAAGAGCATA

241 F E S L P P R L
721 TTTGAGAGTCTACCTCCTAGGCTT

PNPLA4, cDNA: EU419877, protein: ACC91737

1 M K R V N L S F A S C G F L G I Y H L G
1 <u>ATG</u>AAACGTGTCAATCTATCATTTGCATCATGTGGGTTTCTGGGTATATACCACTTGGGG

21 A A A A L Y R H G K K L L K V V K D F A
61 GCAGCAGCTGCTCTTTACAGGCATGGTAAGAAGTTACTGAAAGTTGTGAAAGATTTTGCA

41 G A S A G S L A A T V L L A V P E N I E
121 GGAGCTTCTGCAGGATCCCTGGCTGCCACTGTCTTATTAGCAGTACCAGAAAATATAGAG

61 K C Q Q F T Y E F A E E V R K L D F G A
181 AAATGTCAGCAGTTTACCTATGAATTTGCTGAAGAAGTCAGAAAATTGGACTTTGGTGCA

81 V T P G Y D F M K R L R E G I E S I L P
241 GTAACTCCTGGTTACGATTTTATGAAAAGACTTAGGGAAGGCATAGAATCTATTCTTCCT

101 S N A H E I A E N R L Y V S V T N V R N
301 TCTAATGCTCATGAGATAGCTGAGAATCGGCTCTATGTGTCTGTTACTAATGTGAGAAAT

121 G K N Y L F S N F A S R E D L I K V L L
361 GGGAAAAATTACTTGTTCTCAAATTTTGCCTCCAGGGAGGACCTCATTAAGGTCCTGCTA

141 A S S F V P V Y A G M K P V E Y K G E K
421 GCAAGTAGTTTTGTACCAGTATATGCTGGAATGAAGCCAGTCGAGTATAAAGGAGAGAAG

161 W V D G G I T N G L P I L P F G R T I T
481 TGGGTTGATGGTGGCATTACCAATGGCCTTCCTATCTTGCCTTTTGGACGAACGATTACG

181 I S P F S G R L D I C P Q D K G R V D L
541 ATTTCTCCTTTCAGTGGTCGATTAGATATCTGTCCACAAGATAAAGGACGTGTTGATCTG

```
 201   Y  V  K  F  A  K  Q  D  I  M  L  S  L  A  N  L  V  R  F  N
 601   TATGTTAAATTTGCAAAACAAGATATAATGTTGTCTTTGGCCAACCTAGTAAGATTTAAT

 221   Q  A  M  F  P  P  N  Q  E  K  M  E  S  L  Y  Q  N  G  F  D
 661   CAAGCTATGTTTCCACCAAACCAGGAGAAAATGGAATCACTGTACCAAAATGGTTTTGAT

 241   D  A  V  Y  F  L  L  K  E  N  W  F  E  -
 721   GATGCTGTATATTTTTTACTAAAAGAAAACTGGTTTGAATAG
```

PNPLA6 (partial), cDNA: EU419880, protein: ACC91740

```
   1   V  L  G  H  F  E  K  P  L  F  L  E  L  C  K  H  M  V  F  Q
   2   GTGTTGGGACACTTTGAGAAGCCGCTGTTTCTGGAGCTCTGCAAGCACATGGTTTTCCAG

  21   Q  C  Q  Q  G  D  Y  V  F  R  P  G  Q  P  D  T  S  I  Y  V
  62   CAGTGCCAGCAAGGGGATTACGTATTCCGACCCGGGCAGCCCGACACCAGCATCTACGTG

  41   L  Q  D  G  K  L  E  L  L  L  T  E  P  D  G  K  E  T  V  M
 122   CTGCAGGACGGCAAGCTGGAGCTGCTCCTCACCGAGCCGGATGGAAAGGAGACTGTGATG

  61   K  E  V  F  P  G  D  S  V  H  S  L  L  S  I  L  D  V  I  T
 182   AAAGAGGTGTTTCCTGGAGACAGCGTGCACAGCCTGCTCAGCATCCTCGATGTCATCACG

  81   G  H  Q  R  P  Y  R  T  V  C  A  R  A  A  E  D  S  T  V  L
 242   GGCCACCAGCGGCCATACCGTACGGTGTGTGCGCGGGCAGCAGAGGATTCAACGGTTCTC

 101   R  L  P  V  E  A  F  S  A  V  F  E  K  Y  P  E  S  L  V  R
 302   CGTTTGCCAGTTGAAGCCTTTTCAGCTGTCTTTGAGAAGTACCCCGAGAGCCTGGTGCGG

 121   V  V  Q  I  I  M  V  R  L  Q  R  V  T  F  L  A  L  H  N  Y
 362   GTGGTGCAGATCATCATGGTGCGCTTGCAGCGCGTCACCTTCCTGGCACTGCACAATTAC

 141   L  G  L  T  N  E  L  F  S  H  E  M  Q  P  L  R  L  F  P  Q
 422   CTGGGGCTGACGAATGAGCTTTTCAGCCACGAGATGCAGCCACTGCGGCTCTTCCCGCAG

 161   P  S  H  A  T  R  T  S  P  V  R  H  G  K  R  G  L  G  A  A
 482   CCCAGCCACGCCACCCGCACCAGCCCTGTCCGCCATGGCAAGCGGGGGCTCGGCGCTGCT

 181   E  E  G  R  E  R  P  T  E  L  I  E  P  L  K  A  G  V  L  D
 542   GAGGAGGGAAGGGAGAGACCAACGGAGCTGATTGAGCCGCTGAAAGCCGGAGTTCTGGAT

 201   A  P  A  P  P  L  L  S  R  C  I  S  M  P  V  D  I  S  G  I
 602   GCCCCTGCACCACCACTGCTGAGCCGCTGCATCTCCATGCCAGTGGATATCTCTGGCATC

 221   Q  K  G  P  R  S  D  F  D  M  A  Y  E  R  G  R  I  S  V  S
 662   CAGAAGGGTCCCCGCTCTGACTTTGACATGGCCTACGAGCGCGGACGCATCTCAGTATCG

 241   L  Q  E  D  S  T  S  P  L  A  A  F  S  R  S  I  S  H  E  P
 722   CTGCAGGAGGACAGCACCAGCCCCCTGGCTGCCTTCTCTCGGTCCATCTCACACGAGCCC

 261   K  E  R  K  S  V  T  V  E  E  Q  P  S  G  I  Y  R  Y  S  C
 782   AAGGAACGTAAGTCGGTGACGGTGGAGGAGCAGCCATCGGGTATCTACAGGTACAGCTGT

 281   E  D  E  S  A  A  V  D  C  P  F  E  P  Y  Q  G  R  Q  T  S
 842   GAGGACGAGTCGGCCGCGGTGGACTGTCCCTTCGAGCCCTACCAGGGCCGTCAAACCAGC

 301   A  I  F  E  A  A  K  Q  E  L  V  K  L  M  K  V  E  D  P  S
 902   GCCATCTTCGAGGCTGCCAAGCAGGAGTTGGTCAAACTGATGAAGGTGGAGGACCCTTCT

 321   L  L  N  N  R  V  L  L  H  H  A  K  A  G  T  V  I  A  R  Q
 962   CTGCTCAACAACCGTGTCTTGCTTCATCACGCCAAAGCTGGGACGGTTATTGCCCGTCAA

 341   G  D  Q  D  V  S  L  H  F  V  L  W  G  C  L  H  V  Y  Q  R
1022   GGGGACCAGGACGTGAGCCTCCACTTCGTGCTGTGGGGCTGCCTACATGTCTACCAGAGG
```

```
 361   M  I  D  K  A  E  D  V  C  L  F  L  T  Q  P  G  E  M  V  G
1082   ATGATTGACAAGGCCGAAGATGTCTGCCTCTTCCTGACACAGCCTGGTGAGATGGTGGGG

 381   Q  L  A  V  L  T  G  E  P  L  I  F  T  I  K  A  N  R  D  C
1142   CAGCTGGCCGTGCTCACTGGAGAGCCCCTCATCTTCACCATCAAGGCCAACCGAGACTGT

 401   T  F  L  K  I  S  K  S  D  F  Y  E  I  M  R  E  Q  P  S  V
1202   ACCTTCCTCAAGATCTCCAAGTCAGACTTCTACGAGATCATGCGGGAGCAGCCCAGCGTG

 421   V  L  S  V  A  H  T  V  V  T  R  M  S  P  F  V  R  Q  M  D
1262   GTGCTGAGCGTCGCCCACACCGTGGTCACCCGCATGTCACCCTTCGTGCGCCAAATGGAC

 441   F  A  I  D  W  M  A  V  E  A  G  R  A  L  Y  R  Q  G  D  K
1322   TTTGCCATCGATTGGATGGCGGTGGAAGCCGGCCGGGCGCTCTACAGGCAGGGTGACAAG

 461   S  D  C  T  Y  I  A  L  N  G  R  L  R  S  V  I  Q  K  G  S
1382   TCAGACTGCACCTACATCGCGCTCAACGGGCGGCTCCGCTCCGTCATCCAGAAGGGCAGT

 481   G  K  K  E  L  I  G  E  Y  G  R  G  D  L  I  G  V  V  E  A
1442   GGCAAAAAGGAGCTCATTGGGGAGTACGGCCGCGGGGACCTGATTGGCGTGGTGGAAGCA

 501   L  T  R  Q  P  R  A  T  T  V  H  A  V  R  D  T  E  L  A  K
1502   CTTACCCGGCAACCCCGTGCCACCACGGTGCACGCGGTCCGGGACACGGAGCTGGCCAAG

 521   L  P  E  G  T  L  N  N  I  K  R  R  Y  P  Q  V  V  T  R  L
1562   CTTCCCGAAGGCACCTTGAACAACATCAAGCGTCGGTACCCTCAGGTTGTCACCCGTCTC

 541   I  H  L  L  S  Q  K  I  L  G  N  L  Q  Q  L  R  G  P  F  A
1622   ATCCACCTCCTGAGCCAGAAGATCTTGGGAAACCTCCAGCAGCTCCGCGGCCCCTTCGCA

 561   G  S  G  L  G  M  A  S  S  S  E  P  T  N  P  T  S  N  L  S
1682   GGCTCTGGTTTGGGCATGGCCTCCAGCTCGGAGCCCACCAACCCCACCAGCAACCTGTCA

 581   T  V  A  V  L  P  V  C  D  D  V  P  T  A  A  F  T  L  E  L
1742   ACGGTGGCGGTGCTGCCAGTGTGTGACGACGTGCCCACGGCTGCCTTCACCTTGGAGCTC

 601   K  H  A  L  N  A  I  G  P  T  L  L  L  T  S  D  I  I  R  A
1802   AAGCACGCGCTGAATGCCATCGGTCCCACACTGCTCCTCACCAGTGACATCATCCGCGCC

 621   R  L  G  S  S  A  L  D  S  I  H  E  Y  R  L  S  G  W  L  A
1862   CGACTTGGCTCCTCGGCACTGGACAGCATCCATGAGTACCGCCTGTCGGGCTGGCTGGCC

 641   Q  Q  E  D  I  H  R  I  V  L  Y  Q  T  D  C  T  L  T  P  W
1922   CAGCAAGAAGACATCCATCGCATCGTGCTCTACCAAACCGACTGCACTCTGACGCCGTGG

 661   T  V  R  C  I  R  Q  A  D  C  I  L  I  V  G  L  G  D  Q  E
1982   ACCGTGCGCTGCATCCGGCAGGCGGACTGCATCCTCATTGTTGGGTTGGGTGACCAGGAG

 681   P  A  L  G  E  L  E  Q  M  L  E  N  T  A  V  R  A  L  K  Q
2042   CCAGCGCTCGGAGAGCTGGAGCAGATGCTGGAGAACACGGCGGTGCGTGCACTGAAGCAG

 701   L  V  L  L  H  R  E  D  G  P  S  P  S  R  T  V  E  W  L  N
2102   TTGGTTCTCCTGCACCGTGAGGATGGTCCCAGCCCATCGCGCACCGTTGAGTGGCTCAAC

 721   M  R  S  W  C  S  G  H  L  H  I  K  C  P  R  R  V  F  S  R
2162   ATGCGGAGCTGGTGCTCGGGCCACCTGCACATCAAGTGCCCGCGCCGCGTCTTCTCCCGA

 741   R  S  P  A  K  L  R  E  M  Y  E  K  V  F  A  K  S  A  D  R
2222   CGCAGCCCCGCCAAGCTGCGAGAGATGTACGAGAAGGTGTTCGCCAAGAGCGCCGATCGG

 761   H  S  D  F  S  R  L  A  R  V  L  T  G  N  T  I  A  L  V  L
2282   CACAGCGACTTCTCCCGCTTGGCGCGGGTGCTCACCGGCAACACCATCGCCCTCGTTTTG

 781   G  G  G  G  A  R  G  C  S  H  I  G  V  I  K  A  M  E  E  A
```

```
2342  GGTGGCGGCGGAGCCAGGGGCTGCTCCCACATCGGGGTGATCAAGGCGATGGAGGAGGCG

 801   G  I  P  I  D  M  V  G  G  T  S  I  G  S  F  I  G  A  L  Y
2402  GGGATCCCCATCGACATGGTTGGCGGCACCTCCATCGGATCCTTCATCGGCGCGCTGTAC

 821   A  E  E  R  S  A  V  R  T  K  Q  R  A  R  E  W  A  K  C  M
2462  GCTGAGGAGCGCAGCGCCGTGCGCACCAAGCAGCGGGCACGCGAGTGGGCCAAGTGCATG

 841   N  S  V  F  A  T  V  L  D  L  T  Y  P  I  T  S  M  F  S  G
2522  AATTCGGTGTTTGCGACCGTCCTGGACCTCACTTACCCCATCACCTCCATGTTTTCGGGC

 861   S  A  F  N  A  S  I  N  K  V  F  Q  D  K  Q  I  E  D  L  W
2582  TCAGCCTTCAATGCCAGCATCAACAAAGTTTTCCAGGACAAGCAGATCGAGGACCTTTGG

 881   L  P  Y  F  N  V  T  T  D  I  T  A  S  A  M  R  V  H  T  D
2642  CTTCCCTACTTCAACGTCACAACAGACATCACGGCCTCAGCCATGCGGGTGCACACGGAT

 901   G  S  L  W  R  Y  V  R  A  S  M  T  L  S  G  Y  L  P  P  L
2702  GGCTCGCTCTGGCGGTACGTGCGAGCCAGCATGACCCTATCTGGGTACCTGCCACCCCTC

 921   C  D  P  K  D  G  N  L  L  M  D  G  G  Y  I  N  N  L  P  A
2762  TGCGACCCCAAGGACGGCAACTTGCTGATGGACGGGGGTTACATCAACAACCTGCCAGCT

 941   D  I  A  R  N  M  G  A  K  T  V  I  A  I  D  V  G  S  Q  D
2822  GACATCGCCCGCAACATGGGTGCCAAGACGGTGATTGCCATCGACGTGGGCAGCCAGGAC

 961   E  T  D  L  C  N  Y  G  D  S  L  S  G  W  W  L  L  W  K  R
2882  GAGACGGACCTGTGCAACTACGGGGACAGCCTGTCGGGCTGGTGGCTGCTCTGGAAGCGC

 981   L  N  P  W  A  E  K  V  K  V  P  D  M  A  E  I  Q  S  R  L
2942  CTCAACCCTTGGGCTGAAAAAGTCAAGGTGCCCGACATGGCAGAGATCCAGTCGCGGTTG

1001   A  Y  V  S  C  V  R  Q  L  E  V  V  K  S  S  S  Y  C  E  Y
3002  GCGTATGTGTCGTGCGTGCGGCAGCTGGAGGTGGTGAAGTCCAGCTCGTACTGTGAGTAC

1021   I  R  P  P  I  D  R  F  K  T  M  D  F  G  K  F  D  E  I  Y
3062  ATCCGACCCCCGATCGACCGCTTCAAGACGATGGATTTCGGCAAGTTCGATGAGATCTAT
```

PNPLA7, cDNA: EU419881, protein: ACC91741

```
   1   M  E  E  E  G  N  G  T  S  L  E  A  M  P  A  S  G  I  L  Q
   1  ATGGAAGAAGAAGGCAACGGCACCAGCCTTGAGGCCATGCCAGCAAGTGGAATTCTTCAG

  21   F  V  E  D  R  I  Q  S  T  M  L  T  G  I  A  I  G  A  A  V
  61  TTTGTAGAAGACAGAATACAGAGTACTATGCTGACTGGAATTGCCATTGGAGCTGCAGTT

  41   A  L  F  L  I  G  I  V  V  F  V  I  Y  R  R  V  K  Q  S  K
 121  GCACTGTTCCTGATAGGAATTGTTGTGTTTGTCATTTACAGAAGAGTGAAACAATCCAAA

  61   Q  L  Q  P  H  G  P  Q  Y  R  F  R  K  R  D  K  V  M  F  Y
 181  CAACTTCAGCCACATGGTCCTCAATACAGATTTCGCAAGAGAGACAAAGTGATGTTCTAT

  81   G  R  K  I  M  R  K  V  S  T  L  P  N  T  L  V  G  N  T  V
 241  GGGCGGAAGATCATGAGAAAGGTTTCGACTCTTCCGAACACTCTGGTTGGGAACACTGTG

 101   P  R  Q  R  M  R  K  R  A  K  V  L  N  L  A  K  R  I  L  R
 301  CCTCGCCAAAGGATGAGAAAACGGGCCAAAGTTTTAAATTTGGCTAAAAGGATTCTGCGT

 121   I  K  K  E  C  P  T  L  Q  P  K  E  P  P  P  S  L  L  E  A
 361  ATTAAGAAAGAATGTCCAACTCTGCAGCCAAAAGAGCCACCTCCCTCTCTACTAGAGGCA

 141   D  L  T  E  F  D  V  K  N  S  H  L  P  S  E  V  L  Y  M  L
 421  GATCTGACAGAATTTGATGTAAAGAATTCTCATTTGCCGTCGGAAGTCCTATACATGCTT
```

```
 161  K  N  V  R  V  L  G  H  F  E  K  P  L  F  L  E  L  C  K  H
 481  AAAAATGTTAGGGTTCTTGGTCACTTTGAGAAGCCTCTTTTTCTGGAATTGTGCAAGCAC

 181  M  F  F  V  Q  L  H  E  G  E  Y  I  F  R  P  G  Q  L  D  N
 541  ATGTTTTTTGTGCAGTTGCATGAAGGAGAATACATCTTCCGTCCTGGGCAGCTGGATAAC

 201  S  I  Y  V  V  Q  D  G  K  L  E  V  C  V  Q  E  S  D  G  T
 601  AGCATCTATGTTGTGCAAGATGGAAAGCTGGAAGTTTGCGTTCAAGAAAGTGATGGCACA

 221  E  V  I  V  K  E  V  L  A  G  D  S  V  H  S  L  L  S  I  L
 661  GAAGTGATTGTTAAGGAGGTGCTGGCAGGAGACAGTGTCCACAGTCTTCTCAGCATTCTA

 241  D  V  I  T  G  H  P  A  P  Y  K  T  V  S  A  R  A  A  T  P
 721  GATGTCATCACGGGTCACCCTGCTCCATACAAAACAGTCTCAGCCCGGGCAGCCACTCCA

 261  S  N  I  L  R  L  P  A  S  A  F  Q  D  V  F  Q  K  Y  P  E
 781  TCCAATATACTGCGACTTCCAGCAAGTGCCTTTCAAGATGTCTTCCAGAAATACCCTGAA

 281  T  L  V  R  V  V  Q  I  I  M  V  R  L  Q  R  V  T  F  L  A
 841  ACTCTTGTTAGAGTGGTGCAGATCATCATGGTGAGGCTGCAGAGAGTGACATTCCTTGCC

 301  L  H  N  Y  L  G  L  T  T  E  L  F  N  C  Q  N  Q  A  I  P
 901  TTGCACAACTACCTTGGGCTGACTACTGAGCTCTTCAATTGTCAGAACCAAGCCATCCCA

 321  L  V  S  V  A  S  V  T  S  G  G  S  S  S  K  A  A  K  R  Q
 961  CTGGTCTCTGTGGCAAGTGTCACATCTGGAGGAAGTTCAAGTAAAGCAGCAAAAAGACAA

 341  V  S  S  V  S  E  E  E  R  G  E  K  F  E  K  S  G  E  A  S
1021  GTGTCCAGTGTATCAGAGGAGGAGCGTGGAGAGAAGTTTGAGAAATCTGGGGAAGCATCA

 361  E  M  D  T  L  K  N  S  G  T  E  N  P  E  H  I  F  R  R  S
1081  GAAATGGATACATTGAAGAATTCTGGTACAGAAAACCCTGAGCATATTTTCAGGAGAAGC

 381  L  S  S  P  P  S  T  E  T  S  G  T  S  S  G  A  K  S  D  M
1141  CTTTCTTCACCCCCTTCTACAGAGACTTCTGGTACTTCCAGCGGTGCGAAGTCAGACATG

 401  D  M  A  Y  E  R  A  R  V  H  F  S  S  E  E  A  S  C  S  P
1201  GACATGGCATATGAGAGAGCCAGAGTGCACTTCAGCTCAGAAGAGGCTTCTTGCAGTCCT

 421  V  V  G  K  S  I  L  K  K  T  V  T  V  T  R  T  P  S  V  V
1261  GTTGTGGGCAAGTCGATATTGAAGAAGACTGTGACAGTGACAAGAACCCCTTCAGTAGTT

 441  F  R  Y  T  D  C  E  L  T  A  Q  E  S  C  N  G  K  P  T  D
1321  TTCCGTTACACTGATTGTGAGCTGACTGCACAGGAATCCTGTAATGGCAAACCCACCGAT

 461  A  I  F  E  A  A  K  K  D  L  S  T  L  M  K  L  D  D  P  S
1381  GCCATCTTCGAAGCAGCGAAGAAAGACCTTTCAACCCTGATGAAACTTGATGATCCTTCC

 481  L  L  N  D  R  V  T  L  H  Q  V  T  A  G  T  V  L  S  R  Q
1441  CTGCTGAATGACAGAGTGACACTGCATCAGGTTACTGCTGGGACTGTGCTGTCAAGGCAG

 501  G  D  Q  D  V  N  V  C  F  V  V  S  G  M  L  H  V  Y  Q  Q
1501  GGAGATCAGGATGTTAATGTTTGCTTTGTGGTCTCCGGGATGCTTCACGTGTACCAACAG

 521  K  V  D  S  E  E  D  T  C  L  F  I  T  H  P  G  E  L  V  G
1561  AAGGTCGACTCCGAGGAGGACACCTGCCTGTTCATTACTCACCCAGGAGAACTCGTGGGC

 541  Q  L  A  V  L  T  G  E  P  L  I  F  T  I  K  A  N  R  D  C
1621  CAGCTTGCTGTTCTCACTGGGGAACCACTGATTTTCACCATCAAGGCCAATCGAGACTGT

 561  S  F  L  S  I  S  K  S  H  F  Y  E  I  M  R  E  Q  P  S  V
1681  AGCTTCCTGTCTATTTCCAAGTCCCACTTCTACGAGATAATGCGAGAGCAGCCAAGTGTT

 581  V  L  G  V  A  H  T  V  V  K  R  M  S  S  F  V  R  Q  I  D
1741  GTGCTTGGTGTGGCCCACACTGTTGTAAAGAGAATGTCCTCATTTGTGAGGCAAATTGAC
```

601 F A L D W M E V E A G R A V Y R Q G D K

1801 TTTGCCCTGGATTGGATGGAAGTTGAGGCTGGACGAGCTGTATACAGGCAGGGTGACAAG

621 S D C T Y I V L N G R L R S V I R M D D

1861 TCAGATTGCACATACATCGTCTTGAATGGACGACTGCGCTCAGTCATACGGATGGATGAT

641 G K K H L T G E Y G R G D L I G V V E A

1921 GGGAAGAAGCACCTGACTGGTGAATATGGCCGAGGAGACTTAATTGGAGTGGTAGAGGCA

661 L T H Q P R A T T V H A V R D S E L A K

1981 CTAACCCATCAGCCTAGAGCCACAACAGTTCATGCCGTCAGAGATTCAGAACTTGCCAAA

681 L P E G A L T S I K R K F P Q V V T R L

2041 CTCCCAGAGGGAGCCCTGACATCTATCAAACGCAAATTCCCCCAGGTTGTGACTAGGCTT

701 I H L L G E K I L G S L Q Q G G H P F G

2101 ATTCACTTGCTGGGCGAGAAGATCCTTGGCAGCCTTCAGCAGGGAGGCCATCCTTTTGGA

721 L H S P S S K W D A G N P A S N L S T I

2161 TTGCACAGCCCAAGCAGCAAATGGGATGCCGGAAATCCTGCCAGCAATCTCTCCACAATA

741 A I M P V S E E V P L T A F T L E L K H

2221 GCAATTATGCCGGTGTCTGAAGAGGTGCCACTCACTGCTTTCACTCTGGAGCTCAAGCAT

761 A L S A V G P A L L L T S D N I K Q R L

2281 GCTCTTAGTGCTGTGGGTCCTGCCTTGCTGCTCACAAGTGATAACATCAAACAGCGGCTG

781 G S A A L D S I H E Y R L T S W L G Q Q

2341 GGTTCTGCTGCACTGGACAGTATCCATGAGTACCGCCTGACGAGCTGGCTGGGCCAGCAG

801 E D I H R I V L Y Q A D S T L T P W T Q

2401 GAGGACATCCACCGCATCGTCCTGTACCAGGCAGACAGCACCCTTACTCCGTGGACTCAA

821 R C I R Q A D C I L I V G L G D Q E P T

2461 CGCTGCATCAGGCAGGCTGACTGCATCTTGATAGTGGGACTGGGAGACCAGGAGCCCACT

841 V G E L E R M L E N T A V R A Q K Q L I

2521 GTTGGAGAGCTGGAGAGGATGCTGGAGAACACAGCGGTCCGAGCCCAGAAGCAGCTGATT

861 L L H K E D G P L P S R T V E W L N M R

2581 CTGCTCCATAAGGAGGATGGACCTCTCCCTTCCCGAACTGTGGAATGGCTCAATATGAGG

881 S W C S A H L H L H C P R R V F S K R S

2641 AGCTGGTGTTCTGCTCATCTTCATCTCCACTGTCCACGAAGAGTGTTTTCCAAAAGAAGC

901 L P K L I E M Y E R V F Q K P P D R H S

2701 TTGCCAAAGCTGATAGAGATGTATGAACGTGTGTTCCAGAAGCCACCAGACCGCCACTCG

921 D F S R L A R V L T G N A I A L V L G G

2761 GACTTCTCTCGCCTGGCTCGTGTCCTGACTGGAAATGCCATTGCCCTGGTCCTTGGTGGT

941 G G A R G C S Q V G L I R A L I E A G I

2821 GGGGGAGCCAGGGGGTGCTCCCAGGTCGGGCTTATCAGGGCGCTGATTGAAGCTGGCATC

961 P V D M I G G T S I G A F M S A L Y A E

2881 CCCGTGGATATGATTGGAGGAACGTCCATCGGCGCTTTCATGAGCGCTCTGTACGCAGAA

981 E R S Y N Q M R I K A R Q W A M V M N S

2941 GAACGCAGCTACAATCAGATGAGGATCAAAGCCAGGCAGTGGGCCATGGTTATGAATTCA

1001 V F K T I L D L T Y P I T S M F S G A A

3001 GTGTTTAAGACTATTCTAGACTTGACGTATCCAATAACCTCAATGTTTTCTGGAGCAGCT

1021 F N N S I S N I F K D K Q I E D L W I P

```
3061  TTTAACAACAGCATCAGCAACATCTTCAAGGATAAGCAGATTGAGGATCTGTGGATTCCT

1041   Y  F  T  I  T  T  D  I  T  A  S  A  M  R  V  H  T  D  G  S
3121  TACTTCACAATCACAACTGACATCACTGCCTCAGCAATGAGGGTCCACACCGATGGCTCT

1061   L  W  R  Y  I  R  A  S  M  S  L  S  G  Y  M  P  P  L  C  D
3181  CTGTGGAGGTACATCCGTGCCAGCATGTCTCTCTCTGGGTACATGCCCCCGCTCTGCGAC

1081   P  K  D  G  H  L  L  M  D  G  G  Y  V  N  N  L  P  A  D  V
3241  CCAAAGGATGGGCACCTCCTGATGGATGGAGGTTATGTCAACAACCTGCCTGCTGATGTG

1101   A  R  S  M  G  A  K  V  V  I  A  I  D  V  G  S  R  D  E  T
3301  GCGAGGTCCATGGGTGCAAAGGTGGTGATAGCAATCGATGTGGGGAGCCGGGATGAGACC

1121   N  L  T  N  Y  G  D  C  L  S  G  W  W  L  L  W  K  R  W  N
3361  AACCTCACCAACTACGGTGACTGCTTGTCCGGCTGGTGGCTGCTGTGGAAGAGGTGGAAT

1141   P  L  A  E  K  V  K  V  P  N  M  A  E  I  Q  T  R  L  A  Y
3421  CCACTGGCTGAGAAAGTCAAGGTGCCGAACATGGCAGAGATCCAAACACGGCTCGCGTAC

1161   V  C  C  V  R  Q  L  E  M  V  K  S  S  D  Y  C  E  Y  I  R
3481  GTGTGCTGCGTGCGGCAGCTGGAGATGGTGAAGAGCAGCGACTACTGCGAGTACATCCGG

1181   P  P  I  D  R  Y  G  T  L  D  F  G  K  F  D  E  I  C  E  V
3541  CCACCCATCGACCGCTACGGCACGCTGGATTTTGGGAAGTTTGATGAGATCTGTGAAGTG

1201   G  Y  Q  H  G  K  T  V  F  G  V  W  S  R  S  G  V  L  D  K
3601  GGATATCAACATGGGAAGACTGTGTTTGGTGTCTGGTCCAGGAGCGGCGTCCTTGACAAG

1221   M  L  R  D  R  Q  E  T  H  K  T  K  T  S  D  N  V  T  Y  P
3661  ATGCTACGAGACAGACAAGAGACACACAAAACCAAAACTTCTGATAACGTGACCTATCCC

1241   S  T  S  F  T  D  L  A  E  I  V  S  R  I  E  P  V  K  A  A
3721  AGCACCTCCTTCACAGACCTGGCAGAAATCGTGTCCAGGATCGAGCCAGTGAAAGCTGCC

1261   V  A  D  D  E  S  D  Y  Q  T  E  Y  E  E  E  V  L  D  N  Q
3781  GTGGCTGATGATGAGTCAGACTACCAGACTGAGTATGAGGAAGAAGTTCTGGACAACCAG

1281   K  D  D  Y  I  H  F  I  G  N  Q  A  E  D  D  S  D  S  D  E
3841  AAGGATGATTATATTCATTTCATAGGGAACCAGGCTGAAGACGACTCTGACTCGGATGAA

1301   D  V  Q  I  R  K  R  K  N  L  P  K  V  D  C  Q  A  S  S  T
3901  GATGTACAGATAAGAAAGCGCAAGAATCTCCCAAAGGTGGACTGCCAGGCAAGCAGCACA

1321   G  E  P  A  -
3961  GGAGAGCCAGCCTAG
```

PNPLA8, cDNA: EU419878, protein: ACC91738

```
   1   M  T  V  H  L  S  L  D  A  Y  L  F  F  V  I  N  P  K  S  L
   1  ATGACAGTTCATTTGTCTCTAGATGCATATTTGTTCTTTGTGATTAACCCAAAAAGTCTT

  21   W  W  K  Q  R  N  K  Y  L  C  L  Y  R  P  K  V  C  W  R  I
  61  TGGTGGAAGCAAAGAAACAAGTACCTGTGTTTGTATAGGCCTAAAGTCTGCTGGAGGATA

  41   S  H  A  I  H  L  R  V  F  H  T  G  E  P  Q  Y  K  W  A  R
 121  AGTCATGCTATTCATCTTAGAGTTTTTCATACCGGTGAGCCTCAGTACAAATGGGCCAGA

  61   S  R  T  L  W  Y  S  K  H  I  F  S  R  D  F  L  Y  Y  R  V
 181  AGCAGAACACTGTGGTATAGCAAGCACATTTTCAGTCGCGACTTTTTGTATTACAGAGTT

  81   S  K  L  L  N  S  S  S  K  G  L  T  K  V  N  N  R  M  S  R
 241  TCTAAACTTTTAAATTCTTCTTCCAAAGGACTTACAAAAGTGAACAATCGCATGTCACGA
```

```
 101  I  K  N  T  I  E  S  V  S  K  A  V  S  G  T  H  S  E  L  V
 301  ATTAAGAATACTATAGAATCTGTTTCAAAGGCAGTGTCTGGCACTCACAGTGAACTGGTT

 121  S  R  I  A  R  L  K  S  H  S  G  T  L  G  K  D  T  K  S  N
 361  TCGCGAATAGCTCGTTTAAAGTCACACTCAGGTACTCTAGGAAAGGATACTAAAAGTAAT

 141  A  D  E  N  N  L  N  V  N  L  E  S  G  K  Q  V  P  D  A  R
 421  GCAGATGAAAATAACCTTAATGTGAATCTGGAAAGTGGTAAACAAGTCCCAGATGCCAGA

 161  T  Q  E  D  C  N  R  G  L  A  A  K  K  S  S  N  T  N  E  N
 481  ACTCAGGAGGATTGCAACAGAGGCCTAGCTGCCAAGAAATCCAGTAACACAAATGAAAAC

 181  L  E  F  V  L  N  S  T  S  G  T  N  Q  D  I  P  E  D  S  A
 541  TTGGAGTTTGTGTTAAACAGTACAAGTGGCACTAATCAAGATATTCCAGAAGATTCAGCA

 201  S  A  K  N  H  L  F  H  I  S  Y  L  S  T  S  F  G  E  T  Y
 601  TCTGCTAAAAACCACCTTTTTCATATAAGCTACCTTTCTACAAGTTTTGGAGAGACTTAT

 221  N  F  V  A  D  H  I  N  W  Y  F  S  N  N  F  V  M  D  Q  E
 661  AACTTTGTTGCTGATCATATCAACTGGTACTTCAGTAATAATTTTGTTATGGATCAAGAG

 241  K  K  K  N  A  L  L  Q  G  S  K  S  E  T  K  L  D  S  S  E
 721  AAAAAGAAGAATGCTTTGTTACAGGGCTCTAAAAGTGAAACGAAGCTTGATTCATCAGAG

 261  N  T  V  I  S  E  E  K  T  V  N  S  A  A  A  L  A  P  E  T
 781  AATACTGTTATTAGTGAAGAAAAGACAGTGAATTCGGCAGCTGCTTTAGCTCCTGAAACA

 281  E  T  E  G  S  E  K  S  N  A  A  L  P  V  S  T  K  K  S  I
 841  GAGACTGAGGGTTCTGAGAAGTCAAATGCTGCTCTTCCAGTTTCCACTAAAAAAAGTATT

 301  A  N  F  L  S  Y  P  S  N  S  V  Q  A  F  V  D  S  Y  I  G
 901  GCAAACTTTCTTTCCTATCCCAGTAACAGTGTACAGGCTTTTGTAGACAGTTACATTGGT

 321  G  L  V  P  K  L  R  S  D  T  K  V  T  A  Q  E  K  S  K  Q
 961  GGATTGGTTCCCAAGTTAAGATCGGATACAAAAGTTACTGCACAGGAGAAGAGTAAACAG

 341  L  E  Q  E  G  S  E  K  D  E  E  D  E  I  K  E  T  K  I  A
1021  CTAGAGCAAGAAGGGTCTGAAAAGGATGAGGAGGACGAAATCAAAGAGACTAAGATTGCA

 361  E  E  R  E  K  H  L  S  L  Q  R  E  K  I  I  A  R  V  S  I
1081  GAAGAGAGGGAAAAACATCTGTCCCTTCAGAGAGAAAAGATCATAGCAAGAGTGAGTATC

 381  D  N  R  T  R  A  L  V  Q  A  L  R  R  S  S  N  R  R  V  C
1141  GATAACAGGACTCGAGCTTTAGTTCAAGCCCTTCGAAGATCCTCAAATCGGAGAGTCTGT

 401  I  S  R  V  E  E  L  T  Y  H  L  L  E  F  P  E  S  R  G  V
1201  ATCAGCAGGGTTGAAGAACTGACTTACCATCTTTTGGAATTTCCGGAGAGCAGAGGAGTT

 421  A  I  K  E  K  I  I  P  C  L  L  R  L  R  Q  A  N  D  E  S
1261  GCAATTAAGGAGAAGATAATCCCGTGCCTACTGCGATTAAGACAAGCAAATGATGAAAGT

 441  L  Q  A  A  V  R  E  T  L  A  I  I  G  Y  T  D  P  V  K  G
1321  CTTCAGGCTGCTGTTAGAGAGACTTTAGCAATAATTGGATATACAGATCCAGTGAAAGGC

 461  W  G  V  R  V  L  A  I  D  G  G  G  T  R  G  L  V  A  L  Q
1381  TGGGGAGTTCGAGTCCTTGCTATTGATGGTGGAGGAACAAGGGGTTTGGTTGCACTTCAA

 481  T  L  R  K  L  E  E  L  T  G  K  P  V  H  H  L  F  D  Y  I
1441  ACGCTACGGAAACTAGAAGAACTTACTGGAAAGCCAGTTCATCATCTTTTTGACTACATT

 501  C  G  V  S  T  G  A  I  L  A  F  M  L  G  L  F  H  I  P  L
1501  TGTGGTGTAAGCACAGGAGCTATATTAGCTTTTATGTTGGGGTTATTCCATATCCCTCTG

 521  D  D  C  E  E  L  Y  H  K  L  G  S  D  V  F  K  Q  N  V  I
1561  GATGATTGTGAAGAACTATATCACAAGTTGGGATCAGATGTTTTTAAGCAGAATGTTATT
```

```
 541  V G T V K M G W S H A F Y D S D I W E K
1621  GTTGGAACAGTTAAGATGGGCTGGAGCCATGCCTTTTATGACAGTGATATCTGGGAAAAA

 561  M L K E K M G S N L M I E T A R N S K C
1681  ATGCTCAAAGAAAAAATGGGCTCAAATCTAATGATTGAAACTGCACGAAATTCCAAATGT

 581  P K V A A V S T I V N R G T P L K A F V
1741  CCAAAGGTAGCTGCAGTAAGCACCATTGTGAACAGAGGAACACCACTGAAAGCATTTGTT

 601  F R N Y N H F P G V K S H Y I G G C Q Y
1801  TTTAGAAACTACAATCACTTCCCTGGGGTTAAGTCTCATTATATTGGAGGCTGTCAGTAT

 621  K L W Q A I R A S S A A P G Y F Q E Y V
1861  AAACTGTGGCAGGCCATTAGAGCATCATCTGCTGCACCAGGGTACTTCCAGGAATATGTT

 641  L G N D L H Q D G G L L L N N P S A L A
1921  TTGGGCAATGATCTTCATCAGGATGGAGGCTTGCTTCTAAATAATCCTTCTGCACTGGCA

 661  V H E C K C L W P D V P L Q C L V S L G
1981  GTTCACGAGTGCAAGTGTCTTTGGCCAGATGTTCCACTGCAGTGTCTGGTATCGCTGGGT

 681  T G R Y E S A G K T N V T Y T S L K A K
2041  ACTGGCCGATATGAAAGTGCAGGGAAGACAAATGTCACATACACCAGTTTGAAAGCCAAA

 701  L T N V I S S A T D T E E V H T M L D A
2101  CTCACAAACGTTATCAGCAGTGCAACTGACACAGAAGAAGTTCACACCATGCTGGATGCA

 721  L L P P D T Y F R F N P L M H E D I P L
2161  CTGTTACCGCCAGACACTTATTTCAGATTTAACCCTCTGATGCATGAAGACATACCTCTG

 741  D E S R K E K L S Q L Q T D G I R Y L E
2221  GACGAAAGTCGGAAAGAGAAACTCAGTCAGCTGCAGACGGATGGAATTCGCTATTTAGAA

 761  R N E E K L R K A A K I L T Q E K S T L
2281  CGAAATGAAGAAAAACTGAGAAAAGCTGCAAAGATATTAACACAAGAAAAATCAACTTTA

 781  Q K F H D W I K L K A D M Y E G F P F L
2341  CAGAAATTTCATGATTGGATAAAATTAAAGGCTGATATGTATGAAGGGTTTCCTTTTCTT

 801  S K L -
2401  TCCAAATTGTGA
```

PNPLA9, cDNA: EU419879, protein: ACC91739

```
   1  M Q F L G R L L S Q A F S N P Y R V K E
   1  ATGCAGTTCCTCGGGCGGCTTTTGAGCCAGGCGTTCAGCAACCCGTACCGGGTGAAGGAG

  21  V S A A E Y S G Q S R L T E D G R V R L
  61  GTGTCCGCCGCCGAGTACTCGGGGCAGAGCCGCCTGACGGAGGACGGCCGGGTGCGGCTC

  41  Y G N G R C W D G V L V N P Q S P G V A
 121  TACGGGAACGGCCGCTGCTGGGACGGCGTGCTGGTCAACCCCCAGAGCCCGGGGGTGGCC

  61  F R L F H L D D E A E A L V L F E R Y A
 181  TTCCGGCTCTTCCACCTCGATGATGAAGCGGAAGCCCTGGTGCTCTTTGAGCGCTATGCG

  81  G H L R P F Y E S S A Q P L S L E V L Q
 241  GGCCACCTGCGGCCCTTCTACGAGAGCTCAGCCCAGCCCCTGTCGCTGGAAGTTCTGCAG

 101  Q L T D C V R N H P S W S P A H V A V E
 301  CAGCTCACTGACTGCGTCCGCAACCACCCCAGCTGGTCCCCGGCACACGTAGCGGTGGAG

 121  V G L R E T F R H N R V L S C V N S T D
```

361 GTTGGCCTGCGTGAGACCTTCCGGCACAACCGTGTGCTCAGCTGTGTGAATAGCACGGAC

141 S E D G C T P L H L A C R K G D M A C L
421 AGTGAGGATGGCTGCACCCCGCTGCACCTGGCGTGCCGCAAGGGAGACATGGCCTGCCTG

161 Q E L L E C H A R V D I T D R K G E T V
481 CAGGAGCTGCTGGAGTGCCACGCGCGAGTGGATATCACAGACAGGAAAGGAGAGACGGTT

181 F H Y A V R G S N P Y I I E L L S S A P
541 TTCCACTATGCTGTGCGAGGAAGCAACCCCTATATCATTGAGCTCCTGAGCAGTGCCCCG

201 T V G V N H L S H E G L T P L Y L A C Q
601 ACTGTAGGTGTGAACCACCTGAGCCACGAAGGGTTGACCCCTCTGTATCTCGCTTGTCAG

221 L G K E D M V R S L L K C R A S C N V M
661 CTGGGCAAAGAGGACATGGTTCGGTCTCTGCTGAAGTGCCGTGCCAGCTGTAATGTCATG

241 G T V G Y P I H A A L K Y S Q K G C A Q
721 GGGACGGTGGGCTACCCCATCCATGCAGCTCTGAAGTACTCCCAGAAGGGGTGTGCCCAG

261 A I L E V D A S Q V H S R D P R H E A V
781 GCCATCCTCGAGGTGGATGCCAGCCAGGTCCACTCCAGGGACCCACGCCATGAAGCCGTC

281 P L H W A K K A E M T Q L L L K Y G S E
841 CCGCTGCACTGGGCGAAGAAGGCAGAGATGACGCAGCTGCTGCTTAAGTATGGCTCTGAG

301 V N L T S R T A D M A L H I A V K R G R
901 GTGAACCTGACCAGCCGGACAGCTGACATGGCCCTGCACATTGCAGTCAAGAGAGGTCGT

321 F D C A M V L L T H G A D T N A K G Q D
961 TTTGACTGTGCCATGGTACTGCTGACACACGGGGCTGACACCAATGCCAAGGGCCAGGAT

341 G N T P L H L A M K H D H L D M I K A I
1021 GGCAACACACCACTGCATCTGGCCATGAAGCATGACCACCTGGATATGATCAAAGCAATT

361 V V F G G D V E I P N D F G E T P G L L
1081 GTTGTGTTCGGAGGAGATGTTGAGATCCCTAATGATTTCGGGGAGACGCCGGGGCTGCTG

381 A A R N S K G A N R K V L L D L L Q T V
1141 GCAGCCAGGAACAGCAAAGGTGCGAACCGGAAAGTGCTCTTAGACCTGCTGCAAACTGTA

401 G T E R C L P T I P A S Q S L A P S A A
1201 GGGACCGAACGCTGCCTCCCAACCATTCCTGCCTCCCAAAGTCTGGCACCATCTGCCGCC

421 S F L E G Q P S P R S S S N S L G Y K D
1261 TCCTTCCTGGAAGGCCAACCTTCCCCGCGGAGCAGCTCCAACAGCTTAGGGTACAAGGAC

441 L L Y V S A T L G Q F L K A P D V V D S
1321 CTTCTGTATGTTTCTGCGACACTTGGGCAGTTCTTGAAGGCACCAGATGTTGTGGACTCA

461 P R E G E R N Y D R L L C L D G G G I R
1381 CCCAGAGAGGGTGAGAGAAACTATGACCGTCTCCTGTGTCTGGATGGAGGGGGCATCCGA

481 G L V L I Q L L L A I E K A A G R P I R
1441 GGCCTGGTGCTCATCCAGCTTCTTCTGGCCATCGAAAAAGCTGCGGGCCGTCCCATCCGT

501 E I F D W I A G T S T G G I L A L A I V
1501 GAGATCTTTGACTGGATCGCGGGGACCAGCACTGGGGGCATCTTGGCCTTGGCTATTGTA

521 H G K S M D Y M R C L Y F R M K D M V F
1561 CATGGGAAGTCCATGGACTACATGCGCTGCCTGTACTTCCGCATGAAGGACATGGTGTTC

541 R G S R P Y E S E P L D E F L K K E F G
1621 CGGGGGTCTCGGCCCTACGAGTCAGAGCCCCTGGATGAGTTCTTGAAGAAGGAATTTGGG

561 E N T K M T D V Q K P K V I V T G T L C
1681 GAAAACACCAAAATGACAGATGTCCAAAAACCCAAAGTGATTGTGACAGGGACTCTCTGT

581 D R Q P A E L H L F R N Y P A P E T K I
1741 GACCGACAGCCAGCTGAGCTCCATCTCTTCCGGAATTACCCTGCACCTGAGACAAAAATC

601 S T E Y K T T A T F K P L T Q P E D Q L
1801 TCCACTGAATACAAGACAACTGCGACTTTCAAACCACTCACTCAGCCTGAAGACCAGTTG

621 V W R A A R C S G A A P T Y F R P I G R
1861 GTATGGCGTGCTGCTCGCTGCAGTGGTGCAGCTCCCACTTATTTCCGACCGATAGGGCGC

641 F L D G G L L A N N P T L D A M A E I H
1921 TTTTTGGATGGGGGGCTGCTAGCAAACAACCCAACCCTTGATGCCATGGCGGAGATTCAT

661 E Y N K T L I N K G Q R Q K V R K L G L
1981 GAGTACAACAAGACTCTGATCAACAAGGGTCAGAGGCAGAAAGTAAGAAAATTGGGGTTG

681 V V S L G T G K P P Q V P V S S V D V F
2041 GTGGTCTCCCTGGGGACAGGGAAGCCTCCACAGGTCCCGGTGTCCTCTGTGGATGTCTTC

701 R P T N P W E L A K T V F G A R E L G K
2101 CGCCCCACCAACCCCTGGGAACTGGCAAAGACGGTCTTTGGGGCTAGGGAGCTCGGCAAG

721 M V V D C C T D A D G P A V N R A R A W
2161 ATGGTGGTGGACTGTTGCACTGACGCAGATGGCCCAGCTGTGAATCGTGCCAGGGCCTGG

741 C E M T D I P Y F R L S P Q L H T D V M
2221 TGTGAGATGACAGATATCCCATATTTCCGGCTCAGCCCTCAGCTGCACACAGATGTGATG

761 L D E V N D S V L V N A L W D T Q L Y I
2281 CTGGACGAGGTGAATGACAGCGTGCTGGTGAACGCCCTCTGGGACACCCAGCTCTACATC

781 Y Q Q R E Q L E R L V Q Y L C R -
2341 TACCAGCAGCGGGAGCAGTTGGAGCGGCTGGTGCAGTATCTCTGCCGATGA

CGI-58: (lipase motif indicated in bold), cDNA: EU419873, protein: ACC91733

1 M A E E E A S S E R L G W L F S W L P A
1 ATGGCCGAGGAGGAGGCCTCCAGCGAAAGGTTAGGATGGTTGTTCAGCTGGCTTCCTGCT

21 W C P T S L L H L K E A E D K M L K C I
61 TGGTGCCCCACGTCACTACTGCACCTTAAAGAAGCTGAGGACAAAATGCTAAAATGTATT

41 T S T Y N K R Y V Y L A N G N K I W T L
121 ACAAGCACATACAATAAGCGGTATGTGTATCTAGCTAATGGAAACAAGATATGGACACTG

61 T F S P D L S R K T P L V L L H G F G G
181 ACGTTCTCTCCAGACCTTTCACGTAAAACTCCACTTGTTCTGCTTCATGGATTTGGAGGA

81 G V G M W A L N F E E L C E N R T V H A
241 GGTGTTGGAATGTGGGCTCTCAATTTTGAAGAGCTCTGTGAAAACAGGACCGTTCATGCT

101 F D L L G F G R S S R P H F D T D A R E
301 TTCGACCTCTTGGGATTTGGACGAAGCAGTAGACCACACTTTGATACTGATGCTCGGGAA

121 A E N Q F V E S I E E W R K E M G L E K
361 GCAGAAAATCAGTTTGTGGAATCCATAGAAGAGTGGAGAAAGGAGATGGGGCTAGAAAAA

141 M I **L L G H N L G G** F L A A A Y S L K Y
421 ATGATTTTGCTTGGACACAATCTGGGTGGATTCCTGGCTGCTGCTTACTCGTTAAAATAC

161 P S R V K H L I L V E P W G F P E R P D
481 CCATCAAGGGTCAAACATCTTATCTTAGTGGAGCCATGGGGTTTTCCAGAAAGGCCTGAC

```
 181  N  A  E  H  E  R  P  I  P  I  W  I  K  A  L  G  A  I  L  S
 541  AATGCTGAACACGAAAGACCAATTCCAATCTGGATCAAAGCACTAGGAGCTATACTGAGT

 201  P  F  N  P  L  A  G  L  R  I  A  G  P  F  G  L  S  L  V  Q
 601  CCATTTAATCCATTAGCTGGGCTGAGGATAGCAGGACCCTTTGGTCTAAGCCTTGTTCAG

 221  R  L  R  P  D  F  K  R  K  Y  A  S  M  F  D  D  N  T  V  T
 661  CGTTTAAGACCAGATTTCAAACGAAAATATGCATCGATGTTTGATGATAACACTGTGACT

 241  E  Y  I  Y  H  C  N  V  Q  S  P  S  G  E  T  A  F  K  N  M
 721  GAATATATCTATCACTGCAATGTGCAGTCGCCCAGTGGTGAAACAGCTTTCAAGAACATG

 261  T  I  P  Y  G  W  A  K  R  P  M  L  Q  R  I  P  Q  M  D  Q
 781  ACTATTCCTTATGGATGGGCAAAAAGGCCAATGCTGCAACGGATTCCACAAATGGATCAA

 281  D  I  P  I  T  V  V  Y  G  A  R  S  C  I  D  G  N  S  G  S
 841  GACATTCCTATCACCGTGGTTTATGGAGCACGTTCATGCATAGACGGCAATTCTGGCAGC

 301  T  I  Q  S  L  R  P  K  S  Y  V  K  T  I  A  I  L  G  A  G
 901  ACTATCCAGTCTCTGAGACCAAAGTCATATGTGAAGACAATAGCTATCCTTGGTGCAGGT

 321  H  Y  V  Y  A  D  Q  P  E  D  F  N  Q  K  V  K  D  I  C  D
 961  CATTACGTGTACGCTGATCAGCCTGAAGACTTCAATCAGAAAGTGAAAGACATCTGTGAT

 341  S  V  D  -
1021  TCTGTGGACTGA
```
